# Associations between lifestyle interventions during pregnancy and childhood weight and growth: a systematic review and meta-analysis

**DOI:** 10.1186/s12966-020-01075-7

**Published:** 2021-01-07

**Authors:** Roxana Raab, Sophie Michel, Julia Günther, Julia Hoffmann, Lynne Stecher, Hans Hauner

**Affiliations:** Institute of Nutritional Medicine, Else Kroener-Fresenius-Centre for Nutritional Medicine, School of Medicine, Technical University of Munich, Georg-Brauchle-Ring 62, 80992 Munich, Germany

**Keywords:** Pregnancy, Prenatal lifestyle intervention, Child anthropometry, Childhood obesity

## Abstract

**Background:**

Maternal health and lifestyle during pregnancy may be critical for the onset and progression of childhood obesity. Prenatal lifestyle interventions have been shown to positively affect maternal behaviors, gestational weight gain, and anthropometric outcomes in infants at birth. The influence of such interventions on child weight or growth beyond birth is unknown. We therefore examined the association between lifestyle interventions during pregnancy and anthropometric outcomes during childhood.

**Methods:**

A systematic literature search was conducted in three electronic databases, two clinical trial registers and further sources, without language or publication status restrictions. Additionally, 110 study authors were contacted to obtain unpublished data. Randomized controlled trials comparing any antenatal lifestyle or behavioral intervention to standard prenatal care, in women of any body mass index (BMI), with offspring anthropometric data at 1 month of age or older, were considered. Two reviewers independently extracted data and assessed the risk of bias using the Cochrane Collaboration’s updated tool. Data on weight, length, and BMI, and corresponding z-scores, were stratified into six age ranges and weighted mean differences (WMD) with 95% confidence intervals (CI) were calculated in univariate and multivariate random-effects meta-analytical models.

**Results:**

Twenty trials comprising 11,385 women were included in this systematic review, of which 19 were combined in meta-analyses. Overall, lifestyle interventions during pregnancy were not associated with differences in weight, length, BMI, or corresponding z-scores, in children aged 1 month to 7 years (e.g. weight in 5 to 6 month old children, WMD: 0.02 kg; 95% CI: − 0.05 to 0.10 kg, *I*^*2*^ = 38%; 13 studies, 6667 participants). Findings remained consistent when studies were stratified by maternal baseline BMI or other risk factors, and intervention content and duration. Based on the GRADE criteria, the strength of the body of evidence was considered moderate.

**Conclusion:**

Prenatal lifestyle interventions were not shown to influence childhood weight or growth. Nevertheless, women should be encouraged to pursue a healthy lifestyle during pregnancy. Further efforts to establish early prevention strategies for childhood obesity are urgently needed. Thus, large, high-quality studies with pre-planned, long-term follow-ups are warranted.

**Trial registration:**

PROSPERO CRD42018118678.

**Supplementary Information:**

The online version contains supplementary material available at 10.1186/s12966-020-01075-7.

## Introduction

Childhood obesity is one of the most serious public health concerns worldwide, with prevalence rates steadily increasing over the last decades [1]. Children with overweight or obesity are prone to track excess body weight into adulthood [2], and to face both immediate and long-term physical and psychological health consequences [1, 3, 4]. Hazardous weight and growth patterns may develop as early as in utero or in infancy. Experimental animal studies and observational studies in humans have shown intrauterine exposure to certain conditions, such as maternal obesity, excessive gestational weight gain (GWG), or an unhealthy lifestyle to shape an obesogenic environment for the fetus, and to thereby modify the fetal metabolism [5–11]. This may increase the risk of being born with a high birth weight, large for gestational age (LGA), or for accelerated weight gain during infancy [12–14]. Meta-Analyses and large observational studies with long-term follow-ups have shown both maternal obesity and excessive GWG, as well as anthropometric markers in infancy, to be major risk factors for obesity in child- and adulthood [15–22].

Therefore, early prevention strategies targeting modifiable risk factors for childhood obesity are urgently needed. The World Health Organization’s (WHO) commission on ending childhood obesity has emphasized the management and guidance on appropriate GWG, healthy nutrition, and physical activity during the pre-conceptual and prenatal period as important contributors to the prevention of childhood obesity [23]. The short-term impact of lifestyle interventions during pregnancy has been extensively studied. Systematic reviews and meta-analyses provide evidence for a moderately beneficial intervention effect on maternal outcomes, such as a reduction in excessive GWG [24–26]. Some also point to a reduced risk of LGA and high birth weight [24, 27, 28]. Thus, lifestyle interventions during pregnancy may be able to improve established risk factors for childhood obesity during the prenatal and neonatal period. However, their direct impact on anthropometric outcomes beyond the neonatal period remains unclear. So far, no systematic review with meta-analysis on this topic has been performed. The main reasons were the small amounts of available data and the high variability in reported anthropometric outcomes [29–32]. It is therefore warranted to assess whether such interventions can also improve obesity-related outcomes in children in the longer term. Our primary objective was to assess the association between lifestyle interventions in pregnancy and weight or growth in childhood. Moreover, we endeavored to explore the role of maternal baseline body mass index (BMI) or further risk factors, as well as the role of intervention content and duration through subgroup analyses.

## Methods

This systematic review and meta-analysis followed a pre-specified protocol (PROSPERO CRD42018118678), was based on the methods of the Cochrane Handbook for Systematic Reviews of Interventions [33], and adhered to the Preferred Reporting Items for Systematic Reviews and Meta-Analyses (PRISMA) guidelines (Additional file 1: Material S1) [34].

### Eligibility criteria

Individual-, cluster-, and quasi-randomized controlled trials (RCTs) assessing the effect of any lifestyle intervention in pregnancy, such as diet, physical activity, or mixed interventions, on maternal or offspring weight-related outcomes were considered. Participants were eligible if they had singleton pregnancies, were of any BMI category, and had no serious medical conditions at baseline. Standard prenatal care or minimal intervention groups were accepted as controls. Studies with child anthropometric data at 1 month of age or older were included.

### Data sources and search strategy

On 17 January 2019, the electronic databases PubMed, Embase, the Cochrane Central Register of Controlled Trials (CENTRAL), and the Cochrane Database of Systematic Reviews (CDSR) were systematically searched without language or publication status restrictions for literature published from January 1990 onward (see Additional file 1: Material S2 for exemplary search strategies). In addition, reviewers systematically searched trial registers (the WHO’s meta-register ICTRP and ClinicalTrials.gov) for planned, ongoing, and completed studies on 17 June 2019. Further, they considered grey literature citations, and reference lists of similar systematic reviews and of journal articles of included studies. Internet searches were periodically performed using general search engines up until March 2020, to identify additional follow-ups of included studies, as well as new studies.

### Study selection and data extraction

Both study selection and data extraction were performed by two reviewers independently (RR and SM). Discrepancies were resolved by discussing or by further reviewers (JG or JH). For study selection, titles, abstracts, and full texts or full trial register entries were scrutinized for eligibility. Data on population, intervention, control, outcome and study design (PICOS) characteristics were extracted using a pilot-tested and modified version of a data collection form for intervention reviews from the Cochrane Collaboration [33]. The primary outcomes were absolute child weight, length, and BMI, and secondary outcomes included corresponding z-scores and further anthropometric variables. Study authors were contacted when pertinent data were not reported. Moreover, authors of eligible study protocols, trial register entries, or grey literature references were contacted to obtain unpublished outcome data from ongoing or completed studies. Finally, authors of all studies of lifestyle interventions during pregnancy with a minimum follow-up of 1 month were contacted, whenever maternal anthropometric outcomes or any offspring outcomes were reported.

### Risk of bias and GRADE assessment

Two reviewers (RR and SM) assessed the risk of bias independently. Further reviewers (JG or JH) were consulted to adjudicate unresolved disagreements. The risk of bias in included studies was assessed using the Cochrane Collaboration’s revised tool [35]. Judgements are classified as “low risk of bias”, “some concerns”, and “high risk of bias”. This review considered assignment to interventions, rather than adherence to interventions. The Outcome Reporting Bias in Trials (ORBIT) classification system was used to help identify selective outcome reporting [36]. The quality of the body of evidence was evaluated based on the criteria of the Grading of Recommendations, Assessment, Development and Evaluation (GRADE) system [33]: directness of the evidence, within-study risk of bias, precision of the effect estimate, heterogeneity, and risk of publication bias. The potential for publication bias was investigated by visually assessing funnel plots and performing Egger’s test [37].

### Statistical analyses

Data reported according to the intention to treat principle were extracted. Adjusted or imputed data were only used if unadjusted data could not be obtained. Z-scores were extracted as reported. If outcome data were reported stratified for subgroups, data were pooled using a Cochrane-endorsed formula [33]. Given expected levels of heterogeneity, differences in means between intervention and control groups were pooled in random-effects meta-analysis models, to calculate the weighted mean differences (WMD) with 95% confidence intervals (CI) [33]. Data of individually- and cluster-RCTs were pooled without statistical adjustments, as an interaction of the unit of allocation and the intervention effect was considered unlikely. Heterogeneity between studies was assessed using the *I*^*2*^ statistic and interpreted according to the handbook of the Cochrane Collaboration [33].

All primary and secondary outcome data were stratified according to the age of children at follow-up and synthesized in separate meta-analysis models: 1 to 2 months, 3 to 4 months, 5 to 6 months, over 6 to 12 months, over 12 months to under 3 years old, and 3 years and older. If a study involved more than one follow-up within a specified time range, only the longest follow-up was included within that time range, in order to prevent a unit-of-analysis error [33]. The age ranges were selected to make the most comprehensive use of the available data.

Multivariate random-effects meta-analysis modelling of the primary outcomes was performed, combining all available data across the previously stratified age ranges in a single model, separate for each outcome. By taking into account that outcomes measured at multiple time points within a study may be correlated, this method can result in more precise parameter estimates [38]. Variance-covariance matrices were estimated using within-study correlations [39], calculated from individual participant data (IPD) obtained from four included studies [40–43].

Subgroup analyses were conducted to analyze the associations of interventions with absolute child weight, length and BMI, according to population baseline risk (i.e. maternal baseline BMI or further risk factors), intervention duration (pregnancy only, or pregnancy and postpartum period), and intervention content (diet only, physical activity only, or mixed). Tests for subgroup differences were performed in random-effects models. Sensitivity analyses excluding cluster-RCTs and studies at a high risk of bias were conducted.

*P*-values below 0.05 were considered statistically significant. The reviewers used RStudio software version 1.1.447 (RStudio Inc., Boston, MA, USA) including the packages *meta, metafor, and mvmeta* [44].

## Results

### Study selection

**Fig. 1:**
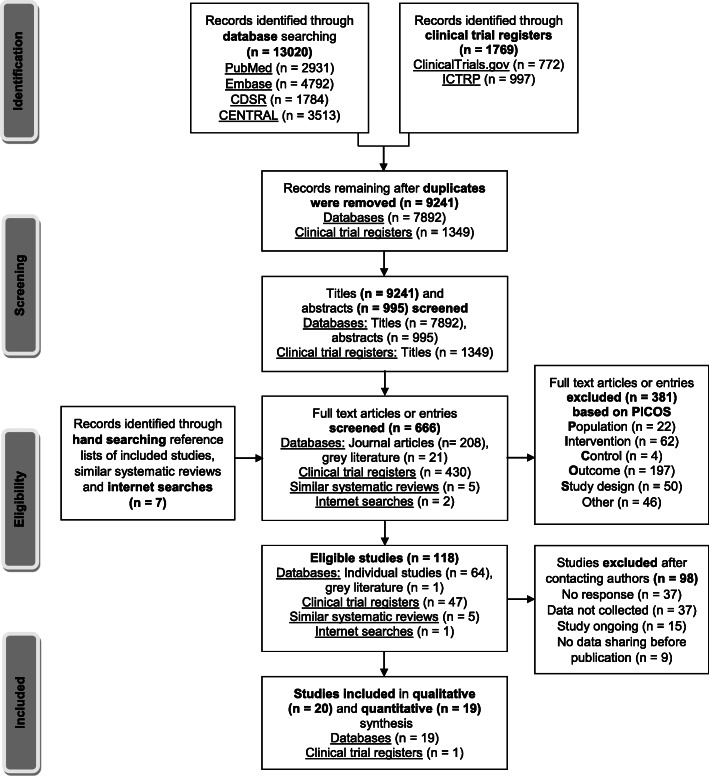
Identification and selection of studies. Abbreviations: PICOS, participant, intervention, control, outcome, study design

Figure 1 depicts the study selection procedure. Full text screening was performed for 666 references. Eight studies with published data on child anthropometric outcomes were included [45–55]. Also, 110 study authors were contacted for unpublished data. Among them, 12 provided study data [40–43, 56–65]. In total, this systematic review included 20 RCTs [40–43, 45–52, 56–63], of which 19 were synthesized in meta-analyses. One study [49] was only included in the qualitative analysis, as available data were not usable for meta-analysis.

### Study and population characteristics

The 17 individually- [41, 42, 45–52, 56–60, 62, 63] and three cluster-RCTs [40, 43, 61] contributed data of 11,385 randomized participants from 11 different countries (Table 1). Sample sizes at randomization ranged from 31 to 2286 participants [43, 60]. Children were followed-up from 1 month to 7 years postpartum. Women of all BMI classes were recruited in eight [40, 43, 45, 56–58, 62, 63], women with overweight or obesity in six [41, 46, 48, 49, 59, 60], and women with obesity in three studies [50–52]. Two studies exclusively included women with risk factors for gestational diabetes mellitus [42, 61], and one study limited eligibility to women who had previously given birth to a macrosomic infant [47]. The majority of studies included predominantly White and well-educated women (Table 1). Three studies were conducted in regions of high socioeconomic deprivation [50, 57, 59]. One of these reported the large majority of participants to be Black, and another recruited mainly women of South Asian origin [57, 59]. Details regarding population and study characteristics can be found in Additional file 1: Table S1.

**Table 1:** Summary of major PICOS characteristics of included studies

Study and country	N randomized (analyzed at last included follow-up)	Last follow-up included in review	BMI class	Population characteristics	Gestational week at inclusion	Intervention duration	Intervention content
**Chiavaroli et al.** [56], **2018** New Zealand	98 (57)	7 y	All	Only nulliparous women aged 20–40, who were relatively sedentary included	< 20	Pregnancy	PA
**Delta Healthy Sprouts** [57] USA	105 (46)	12 m	All	Large majority of women were of African-American race and unmarried, from region with high rates of OB, diabetes, hypertension	< 19	Pregnancy and postpartum	Mixed
**ETIP** [41, 64] Norway	91 (70)	3 m	OW/OB (≥ 28 kg/m^2^)	Only previously sedentary women included	≤ 18	Pregnancy	PA
**ETOIG** [49] France	275 (238)	2 y	OW/OB	Majority of women were found to be of high socioeconomic status and have high levels of education	≤ 21	Pregnancy and postpartum	Mixed
**FeLIPO** [40] cluster-RCT Germany	250 (220)	10–12 m	All, except UW	Majority of women were normal weight, German-born	< 18	Pregnancy	Mixed
**Fit for Delivery** [58] USA	401 (222)	6 m	All (19.8–40 kg/m^2^)	Only non-smokers included, most were non-Hispanic White	10–16	Pregnancy	Mixed
**GeliS** [43] cluster-RCT Germany	2286 (1716)	10–12 m	All (18.5–40 kg/m^2^)	Women were predominantly White, relatively well educated, more nulliparous women in intervention group	< 12	Pregnancy and postpartum	Mixed
**HAPPY** [59] United Kingdom	120 (78)	12 m	OW/OB	Women were mostly of South Asian origin, city characterized by high levels of socioeconomic deprivation and ethnic diversity	10–26	Pregnancy and postpartum	Mixed
**Healthy Mom Zone** [60, 65] USA	31 (19)	5–11 wks	OW/OB (25–45 kg/m^2^)	Most women were married, middle to upper class, Caucasian, from rural/ suburban areas	> 8	Pregnancy	Mixed
**Healthy Moms** [52] USA	118 (103)	12 m	OB	Women were primarily White with at least a high school education. Over half were classified with class 2 or 3 obesity	≤ 20	Pregnancy	Mixed
**Kong et al.** [48]**, 2014** USA	42 (33)	6 m	OW/OB	Only non-exercising non-smokers included, cohort predominantly White, married, educated	< 15	Pregnancy	PA
**LIMIT** [46, 53, 54] Australia	2212 (1418)	3–5 y	OW/OB	Women were predominantly White	10–20	Pregnancy	Mixed
**LiPO** [51] Denmark	360 (157)	2.8 y	OB (30–45 kg/m^2^)	Women were exclusively Caucasian	10–14	Pregnancy	Mixed
**NAMI** [45] Finland	171 (143)	6 m	All	Women were exclusively White, majority had high education levels and were primiparous	< 17	Pregnancy and postpartum	Diet
**NELLI** [61] cluster-RCT Finland	442 (150)	7 y	All	Minimum 1 out of 4 risk factors required: BMI ≥ 25 kg/m^2^, history of GDM/ macrosomic birth, age > 40 y, diabetes in family	8–12	Pregnancy	Mixed
**RADIEL** [42] Finland	728 (320)	5 y	All	History of GDM or BMI ≥ 30 kg/m^2^ were required, women were exclusively White, majority well educated, non-smokers, primiparous	< 20, or planning pregnancy	Pre-or early pregnancy and postpartum	Mixed
**ROLO** [47, 55] Ireland	800 (280)	6 m	All	Only secundigravid women with macrosomic birth included, majority were White	≤ 18	Pregnancy	Diet
**Stafne et al.** [62]**, 2012** Norway	855 (258)	15 m	All	Only White women included, cohort generally exercised regularly, BMI mostly in normal range	18–22	Pregnancy	PA
**UPBEAT** [50] United Kingdom	1555 (677)	6 m	OB	Sample characterized by high ethnic diversity and socioeconomic deprivation; women aged ≥16 y were included	15–18	Pregnancy	Mixed
**VIGA** [63] Sweden	445 (185)	6 y	All (≤ 19 kg/m^2^)	Larger proportion of normal weight women compared to the country population	≤ 16	Pregnancy and postpartum	PA

*Abbreviations*: *BMI* Body mass index, *GDM* Gestational diabetes mellitus, *m* Months, *OB* Obesity, *OW* Overweight, *PA* Physical activity, *RCT* Randomized controlled trial, *UW* Underweight, *wks* Weeks, *y* Years

Thirteen interventions were based on a combination of dietary and physical activity components (Table 1). In five studies, interventions comprised physical activity components only [41, 48, 56, 62, 63], and two solely involved dietary counselling [45, 47]. Materials for monitoring and self-assessment, including weight gain charts, logbooks, and pedometers complemented the intervention in a majority of studies (Additional file 1: Table S2). Thirteen studies delivered interventions in pregnancy only, while seven studies continued intervention sessions postpartum [42, 43, 45, 49, 57, 59, 63]. While comparator groups generally received only country-specific prenatal care, in a few studies, some lifestyle advice or full intervention sessions [49, 50, 52], were provided to controls, also. Further details regarding intervention characteristics can be found in Additional file 1: Table S2.

Outcome data available to assess differed as per Table 2. The most frequently obtained anthropometric data were child weight, length, and BMI, followed by corresponding z-scores (Table 2). While half of the studies initially intended to assess obesity-related outcomes in children beyond birth, all of the studies aimed to address risk factors for childhood obesity (Additional file 1: Table S2). These included maternal and infant variables such as excessive GWG or LGA. A number of studies performed unplanned follow-ups and secondary analyses of infant anthropometric and obesity-related outcomes. Further, anthropometric outcomes, such as total body composition or skinfold thickness were seldomly measured, and reported in various ways, and could therefore not be synthesized in meta-analyses.

**Table 2:** Number of studies with published or received child anthropometric data at minimum 1 month postpartum

Outcome	Number of studies for which data could be obtained from published articles	Number of studies for which data were received from study authors	Total number of studies
**Weight (kg)**	8	11	19
**Length (cm)**	7	10	17
**BMI (kg/m**^**2**^**)**	2	10	12
**Weight-for-age z-score**	6	3	9
**Length-for-age z-score**	5	3	8
**BMI z-score**	4	3	7
**Weight-for-length z-score**	5	1	6
**Overweight/ obesity**	5	1	6
**Waist/ abdominal/ hip circumference**	6	0	6
**Skinfold thickness**	6	0	6
**Body composition**	5	0	5
**Rapid infant weight gain**	3	0	3

### Risk of bias and GRADE assessment

**Fig. 2:**
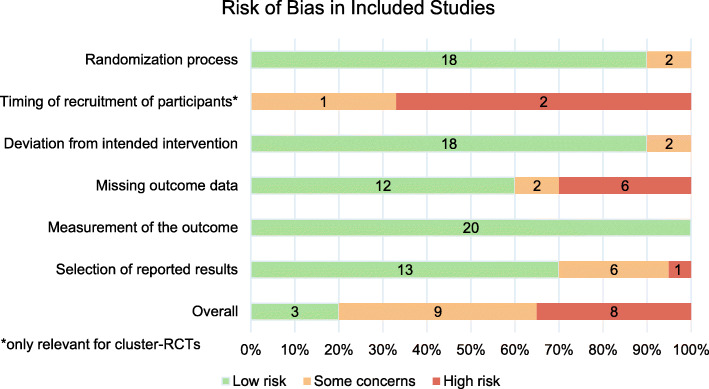
Summary figure of assessment of risk of bias in included studies. Abbreviations: RCT, randomized controlled trial

Figure 2 summarizes the results of the risk of bias assessment for the 20 included studies. Reviewers judged nine studies to be of some concern [41, 43, 45, 48–50, 52, 58, 59], eight to be at a high risk [40, 47, 51, 56, 57, 60–62], and three to be at a low risk of bias across all domains [42, 46, 63]. Missing outcome data were the most frequent reason that studies were judged to be at a high risk of bias [47, 51, 56, 57, 61, 62]. Based on the GRADE criteria, the strength of the body of evidence was considered to be moderate in terms of the outcomes weight, length, and BMI, respectively (Additional file 1: Table and text S3). Seventeen out of 18 funnel plots and Egger’s tests did not indicate publication bias (Additional file 1: Fig. S1).

### Primary outcomes

#### Absolute weight, length, and BMI

**Fig. 3:**
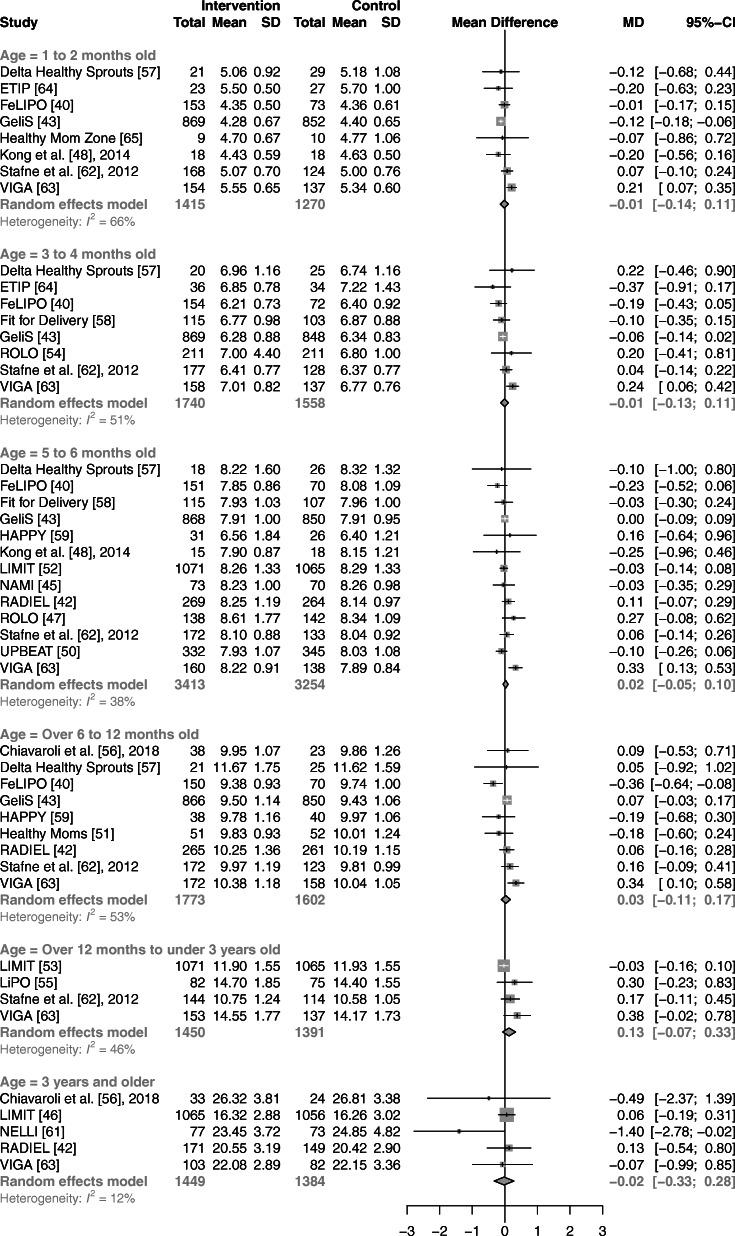
Forest plot illustrating the associations between prenatal lifestyle interventions and child weight in kg. Abbreviations: CI, confidence interval; MD, mean difference; SD, standard deviation

**Fig. 4:**
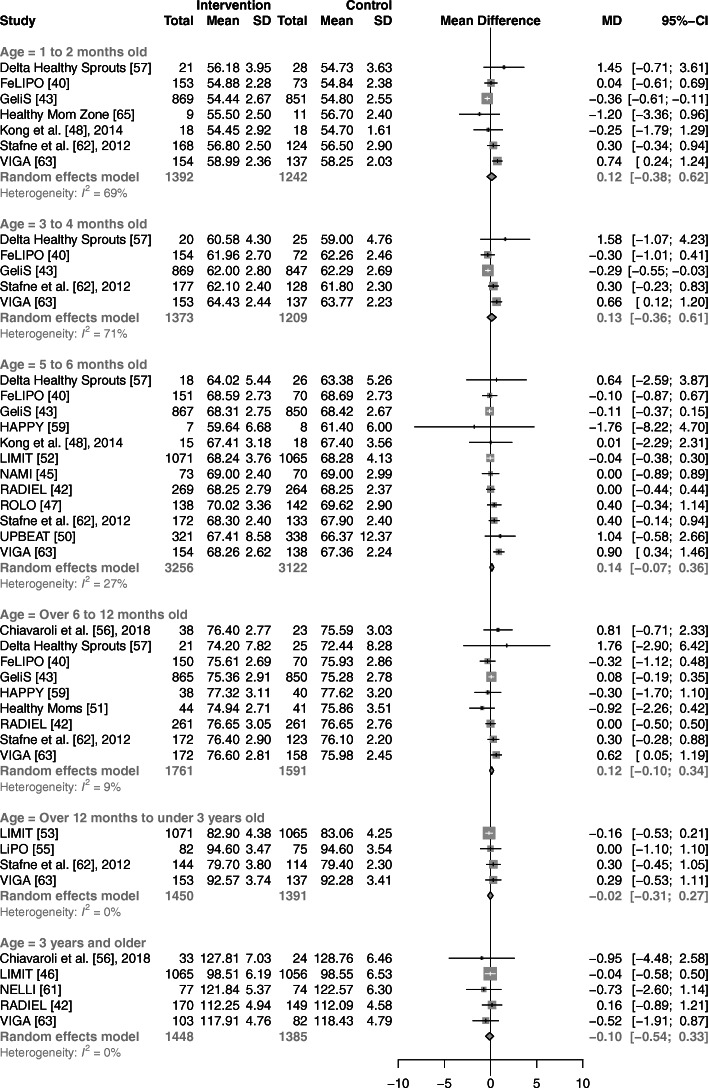
Forest plot illustrating the associations between prenatal lifestyle interventions and child length in cm. Abbreviations: CI, confidence interval; MD, mean difference; SD, standard deviation

**Fig. 5:**
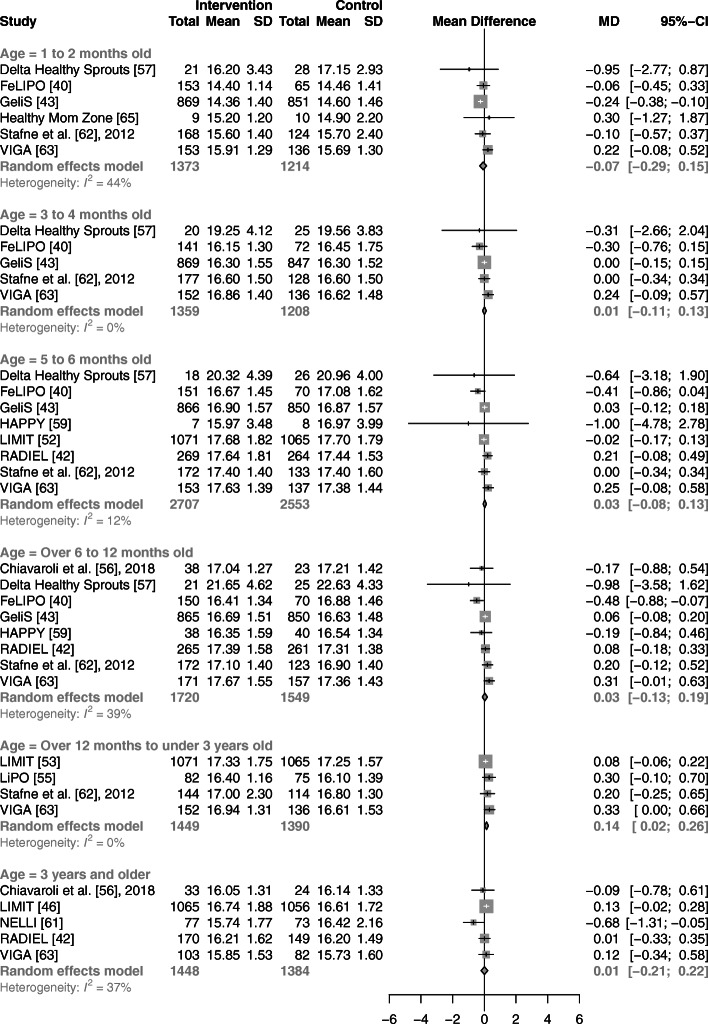
Forest plot illustrating the associations between prenatal lifestyle interventions and child BMI in kg/m^2^. Abbreviations: CI, confidence interval; MD, mean difference; SD, standard deviation

Univariate random-effects meta-analyses showed no association between prenatal lifestyle interventions and changes in body weight or length of children, compared with controls, for any of the six age ranges (Fig. 3, 4). Heterogeneity (*I*^*2*^*)* across the six age categories ranged from 12 to 66% and 0 to 71% for weight and length, respectively. Compared with children of control groups, those of intervention groups showed no significant differences in BMI in five out of six age ranges (Fig. 5). Synthesis of four studies revealed a higher BMI in offspring of the intervention groups for those aged over 12 months to under 3 years (WMD: 0.14 kg/m^2^; 95% CI: 0.02 to 0.26 kg/m^2^, *I*^2^ = 0%). Across all age ranges, heterogeneity varied from 0 to 44%.

### Multivariate random-effects meta-analyses of weight, length, and BMI

Multivariate random-effects meta-analyses of weight, length, and BMI for the six age ranges generally showed no significant differences between children of intervention and control groups (Additional file 1: Table S4). Again, a higher BMI in offspring of intervention groups for those over 12 months to under 3 years old was observed (WMD: 0.14 kg/m^2^; 95% CI: 0.02 to 0.26 kg/m^2^; *I*^*2*^ = 12%).

### Secondary outcomes

#### Weight-for-age, length-for-age, and BMI z-scores

Univariate analyses showed no significant differences in weight-for-age z-score (Additional file 1: Fig. S2A), length-for-age z-score (Additional file 1: Fig. S2B), or BMI z-score (Additional file 1: Fig. S2C), with the exception of children aged 3 years or older. In this age range, BMI z-score was found to be significantly higher in children of intervention groups (WMD: 0.10 ; 95% CI: 0.01 to 0.19 ; *I*^*2*^ = 0%, 3 studies). Heterogeneity ranged from 0 to 53% across all analyses.

### Subgroup analyses of weight, length, and BMI

#### Population baseline risk

When studies were stratified by population risk at baseline, tests for subgroup differences did not show significant results for any of the defined age ranges of any of the primary outcomes (Additional file 1: Table S5A-C).

#### Duration of intervention (pregnancy only, or pregnancy and postpartum)

When studies were stratified according to intervention duration, significant subgroup differences were not observed(Additional file 1: Table S5A-C).

#### Type of intervention (diet only or physical activity only, or mixed)

Testing for subgroup differences by intervention content revealed children of women who received physical activity interventions to be taller across various age ranges (1 to 12 months) (Additional file 1: Table S5B), and heavier at over 6 to 12 months of age (WMD: 0.24 kg vs − 0.06 kg; *P* = 0.01). Conversely, after mixed interventions, children aged 3 to 4 months old were shorter (WMD: − 0.28 cm vs 0.48 cm; *P* = 0.001), and children aged 1 to 2 months old had a lower BMI (WMD: − 0.22 kg/m^2^ vs 0.11 kg/m^2^; *P* = 0.04).

For subgroup analyses, heterogeneity ranged from 0 to 88% (Additional file 1: Table S5A-C).

### Sensitivity analyses

When studies judged to be at a high risk of bias were excluded from meta-analyses, results for weight and length remained consistent (Additional file 1: Table S6A-B). BMI was no longer significantly higher in children of the intervention groups over 12 months to under 3 years old (Additional file 1: Table S6C). In sensitivity analyses excluding cluster-RCTs, results for weight and BMI remained unchanged from the main analyses, while 3 to 4 month old infants of intervention groups appeared to be taller (WMD: 0.50 cm; 95% CI: 0.13 to 0.87 cm; *I*^*2*^ = 0%, 3 studies) (Additional file 1: Table S6A-C).

## Discussion

By synthesizing data from 20 RCTs, we found moderately strong evidence for no association between lifestyle interventions during pregnancy and absolute child weight, length, or BMI, or corresponding z-scores, compared to standard prenatal care. Findings of the univariate analyses were consistent across age ranges and different outcomes, and were generally confirmed by those of the multivariate random-effects meta-analyses and z-score analyses. Absolute BMI and BMI z-scores were slightly higher in children of intervention groups, aged over 12 months to under 3 years, and 3 years and older, respectively. However, when studies considered to be at a high risk of bias were excluded from analyses, BMI was no longer significantly different between groups. Given the small number of combined studies for these age ranges, and that the differences between groups are likely too small to be clinically meaningful, results of absolute BMI and BMI z-score analyses should be interpreted cautiously. A reasonable interpretation of these findings on the physiological level may be that intervention effects were too small to impact the intrauterine environment and fetal metabolism. This could have led to the lack of an observable effect on offspring anthropometry. Moreover, a number of studies reported no significant differences resulting from prenatal lifestyle interventions in terms of GWG, maternal health behaviors, or anthropometric outcomes at birth, as well as low adherence to interventions. This may have prevented any downstream effects on offspring weight or growth trajectories. Additionally, numerous other environmental and individual factors after birth, such as rapid infant weight gain [15, 66], may cumulatively affect weight and growth in children [67], and may be of greater importance than in utero factors. In line with our findings, previous reviews have described a limited impact of lifestyle interventions during pregnancy on childhood anthropometric outcomes [29–32]. However, high variability in reported outcomes and heterogeneity across individual studies precluded the reviews from conducting a meta-analysis. This review also endeavored to analyze the association between antenatal lifestyle interventions and further adiposity measures in children, such as body composition or skinfold thickness. However, data were rarely available and conducting meta-analyses was therefore not possible.

The included trials varied in population, intervention, and other study characteristics. With a few exceptions, subgroup analyses did not suggest differences between groups according to content (diet only, physical activity only, or mixed) or duration of interventions (pregnancy only, or pregnancy and postpartum period). Minor, statistically significant differences between groups were likely too small to be clinically meaningful. Moreover, results of subgroup analyses were often influenced by multiple follow-ups of the same few studies. When studies were stratified by maternal baseline BMI or further risk factors, no differences were observed for child weight, length or BMI, for any age range. In line with this finding, a pooled analysis of seven antenatal lifestyle intervention studies in women with overweight or obesity (i.e. high-risk populations) of the LIFE-Moms consortium observed no differences for any infant anthropometric outcome at 12 months of age [68]. Once published, results of a large ongoing IPD meta-analysis may further add to the evidence on the association between lifestyle interventions in pregnancy and obesity-related outcomes in children of women with overweight or obesity [69].

### Strengths

This is the first systematic review to quantitatively synthesize a comprehensive and comparably large body of evidence (20 studies with over 11,000 participants) on the association between antenatal lifestyle interventions and offspring anthropometry from 1 month up to 7 years of age. This review was extensive in its scope, search strategy, and data collection process, and thus minimized the impact of selection and publication biases. To increase the quality of the body of evidence, only RCTs and quasi-RCTs were included. Studies conducted among women of all BMI categories, and in both general and high-risk populations were included. This broadens the overall applicability of findings. By analyzing childhood anthropometrics in six different age groups, we were able to include multiple follow-ups from individual studies, without introducing a unit of analysis error [33]. Further, multivariate random-effects meta-analyses for each primary outcome were performed [38]. IPD from four included studies were used to estimate correlations between age groups. This allowed the previously stratified follow-ups to be synthesized in one model.

### Limitations

Several study-specific limitations reduced the quality of included RCTs, and therefore, the overall strength of the body of evidence of our analyses. The risk of bias assessment revealed a number of studies to have high proportions of missing outcome data, thus limiting statistical power and introducing potential biases. This may in part be explained by studies performing unplanned follow-ups. Further, we observed variable and selective outcome reporting, as has been previously reported [70]. The broad inclusion criteria of this review may have caused heterogeneity in the PICOS characteristics of included studies. Subgroup analyses to explore heterogeneity were performed, however these often only included small numbers of studies and were therefore underpowered. Also, for a number of studies, primary outcomes were related to pregnancy or childbirth, rather than to offspring anthropometry, which limits the directness of evidence. Further, while crude weight, length, BMI, and corresponding z-scores are accurate indicators of anthropometric trajectories in children, they are generally not considered to be valid measures of childhood adiposity [71]. Analyses of skinfold thickness, waist circumference, or whole-body composition measured for instance via DXA scans may have had the potential to improve the clinical significance of findings, however, only a small number of studies measured or reported these outcomes. Finally, this review examined the effectiveness of assignment to interventions, meaning the intention-to-treat effect. Therefore, adherence to prescribed interventions was not considered.

### Clinical implications of findings

Our findings suggest that lifestyle interventions in pregnancy are unlikely to be associated with offspring anthropometry. Nonetheless, prenatal lifestyle interventions have been shown to beneficially influence maternal obesity-related outcomes [24–26, 72], health behaviors [73–75], as well as neonatal outcomes [24, 27, 28]. Moreover, in line with previous research, findings of this review suggest that lifestyle interventions in pregnancy do not negatively impact weight or growth trajectories in children [24, 26, 27, 76]. Thus, antenatal lifestyle interventions should be continued to be implemented in clinical practice.

### Implications for future research

Future research should focus on generating robust data on the effect of prenatal lifestyle interventions on short- and long-term obesity-related outcomes in children. This requires the design of adequately powered studies with pre-planned, long-term follow-ups. Further efforts to improve participant retention rates are pertinent. In addition, defining a core outcome set for trials assessing obesity-related outcomes in children is essential, as has been previously proposed [29, 70, 77]. Only three interventions targeted socioeconomically disadvantaged populations or ethnic minorities. Thus, more interventions tailored to minorities and low- and middle-income settings are needed to increase the applicability of findings to populations most at risk of childhood obesity.

## Conclusion

While lifestyle interventions in pregnancy may positively influence behavioral and weight-related outcomes in mothers, they were not found to be associated with short- or long-term weight or growth outcomes in children. The effectiveness of various interventions , including more comprehensive approaches targeting the preconception, prenatal and postpartum period should be evaluated in future analyses. However, this will require a greater number of large and high-quality studies with pre-planned follow-ups throughout childhood and consistent reporting of core anthropometric outcomes.

## Supplementary Information

**Additional file 1: Material S1.** Prisma checklist. **Material S2.** Exemplary search strategies. **Table S1.** Detailed summary of study, maternal and infant characteristics. **Table S2.** Intervention characteristics of included studies. **Table S3.** Summary of GRADE assessment results. **Table S4.** Multivariate random-effects meta-analyses of weight, length, and BMI. **Table S5.** Summary of subgroup analyses of (A) weight, (B) length, and (C) BMI. **Table S6.** Summary of sensitivity analyses of (A) weight, (B) length, and (C) BMI. **Figure S1.** Funnel plots of (A-F) weight, (G-L) length, and (M-R) BMI data for the six defined age ranges. **Figure S2.** Forest plots illustrating the association of prenatal lifestyle interventions with (A) weight-for-age, (B) length-for-age, and (C) BMI z-scores in children.

## Data Availability

Individual participant data from four studies with different data sharing policies were used. Restrictions apply to the availability of these data, which were used under license for the current study, and so are not publicly available. Data are however available from the authors upon reasonable request and with written permission of the responsible parties of the individual studies. All other data generated or analyzed during this study are included in this published article and its supplementary information files.

## Abbreviations

GWG: Gestational weight gain
LGA: Large for gestational age
WHO: World Health Organization
BMI: Body mass index
PRISMA: Preferred Reporting Items for Systematic reviews and Meta-Analyses
RCT: Randomized controlled trial
CENTRAL: Cochrane Central Register of Controlled Trials
CDSR: Cochrane Database of Systematic Reviews
RR: Roxana Raab
SM: Sophie Michel
JG: Julia Günther
JH: Julia Hoffmann
PICOS: Participant, intervention, control, outcome, study design
ORBIT: Outcome Reporting Bias in Trials
GRADE: Grading of Recommendations, Assessment, Development and Evaluation
WMD: Weighted mean difference
CI: Confidence interval
IPD: Individual participant data
HH: Hans Hauner
LS: Lynne Stecher
