# Supplementary material for: Associations between lifestyle interventions during pregnancy and childhood weight and growth: a systematic review and meta-analysis
Source: Int J Behav Nutr Phys Act. 2021 Jan 7;18:8. doi: 10.1186/s12966-020-01075-7 (PMC7792105; doi:10.1186/s12966-020-01075-7)
Supplement: Supplementary file 1 — Additional file 1: Material S1. Prisma checklist. Material S2. Exemplary search strategies. Table S1. Detailed summary of study, maternal and infant characteristics. Table S2. Intervention characteristics of included studies. Table S3. Summary of GRADE assessment results. Table S4. Multivariate random-effects meta-analyses of weight, length, and BMI. Table S5. Summary of subgroup analyses of (A) weight, (B) length, and (C) BMI. Table S6. Summary of sensitivity analyses of (A) weight, (B) length, and (C) BMI. Figure S1. Funnel plots of (A-F) weight, (G-L) length, and (M-R) BMI data for the six defined age ranges. Figure S2. Forest plots illustrating the association of prenatal lifestyle interventions with (A) weight-for-age, (B) length-for-age, and (C) BMI z-scores in children. [file 12966_2020_1075_MOESM1_ESM.pdf]

Supporting Information

Associations between lifestyle interventions during pregnancy and childhood weight and growth: a systematic review and meta-analysis

Roxana Raab[1*], Sophie Michel[1*], Julia Günther[1], Julia Hoffmann[1], Lynne Stecher[1], Hans Hauner[1]

*Roxana Raab and Sophie Michel contributed equally and share first authorship.

Affiliation: [1]Institute of Nutritional Medicine, Else Kroener-Fresenius-Centre for Nutritional Medicine, School of Medicine, Technical University of Munich, Georg-Brauchle-Ring 62, 80992 Munich, Germany.

Corresponding author:

Hans Hauner, Institute of Nutritional Medicine, Else Kroener-Fresenius-Centre for Nutritional Medicine, School of Medicine, Technical University of Munich, Georg-Brauchle-Ring 62, 80992 Munich, Germany. Telephone: +49 (0)89 289249-21, fax: +49 (0)89 289249-13, email: hans.hauner@tum.de.

Material S1 Prisma checklist

Section/Topic	*#*	*Checklist Item*	*Reported on Page #*
**TITLE**			
Title	1	Identify the report as a systematic review, meta-analysis, or both.	1
**ABSTRACT**			
Structured summary	2	Provide a structured summary including, as applicable: background; objectives; data sources; study eligibility criteria, participants, and interventions; study appraisal and synthesis methods; results; limitations; conclusions and implications of key findings; systematic review registration number.	4
**INTRODUCTION**			
Rationale	3	Describe the rationale for the review in the context of what is already known.	5
Objectives	4	Provide an explicit statement of questions being addressed with reference to participants, interventions, comparisons, outcomes, and study design (PICOS).	5
**METHODS**			
Protocol and registration	5	Indicate if a review protocol exists, if and where it can be accessed (e.g., Web address), and, if available, provide registration information including registration number.	6
Eligibility criteria	6	Specify study characteristics (e.g., PICOS, length of follow-up) and report characteristics (e.g., years considered, language, publication status) used as criteria for eligibility, giving rationale.	6
Information sources	7	Describe all information sources (e.g., databases with dates of coverage, contact with study authors to identify additional studies) in the search and date last searched.	6-7
Search	8	Present full electronic search strategy for at least one database, including any limits used, such that it could be repeated.	6
Study selection	9	State the process for selecting studies (i.e., screening, eligibility, included in systematic review, and, if applicable, included in the meta-analysis).	7
Data collection process	10	Describe method of data extraction from reports (e.g., piloted forms, independently, in duplicate) and any processes for obtaining and confirming data from investigators.	7

Section/Topic	*#*	*Checklist Item*	*Reported on Page #*
Data items	11	List and define all variables for which data were sought (e.g., PICOS, funding sources) and any assumptions and simplifications made.	7
Risk of bias in individual studies	12	Describe methods used for assessing risk of bias of individual studies (including specification of whether this was done at the study or outcome level), and how this information is to be used in any data synthesis.	7
Summary measures	13	State the principal summary measures (e.g., risk ratio, difference in means).	7-9
Synthesis of results	14	Describe the methods of handling data and combining results of studies, if done, including measures of consistency (e.g., I^2) for each meta-analysis.	7-9
Risk of bias across studies	15	Specify any assessment of risk of bias that may affect the cumulative evidence (e.g., publication bias, selective reporting within studies).	7
Additional analyses	16	Describe methods of additional analyses (e.g., sensitivity or subgroup analyses, meta-regression), if done, indicating which were pre-specified.	8-9
RESULTS			
Study selection	17	Give numbers of studies screened, assessed for eligibility, and included in the review, with reasons for exclusions at each stage, ideally with a flow diagram.	10
Study characteristics	18	For each study, present characteristics for which data were extracted (e.g., study size, PICOS, follow-up period) and provide the citations.	10-11
Risk of bias within studies	19	Present data on risk of bias of each study and, if available, any outcome-level assessment (see Item 12).	11
Results of individual studies	20	For all outcomes considered (benefits or harms), present, for each study: (a) simple summary data for each intervention group and (b) effect estimates and confidence intervals, ideally with a forest plot.	11-12
Synthesis of results	21	Present results of each meta-analysis done, including confidence intervals and measures of consistency.	11-12
Risk of bias across studies	22	Present results of any assessment of risk of bias across studies (see Item 15).	11
Additional analysis	23	Give results of additional analyses, if done (e.g., sensitivity or subgroup analyses, meta-regression).	12-13

Section/Topic	*#*	*Checklist Item*	*Reported on Page #*
DISCUSSION			
Summary of evidence	24	Summarize the main findings including the strength of evidence for each main outcome; consider their relevance to key groups (e.g., health care providers, users, and policy makers).	14-15
Limitations	25	Discuss limitations at study and outcome level (e.g., risk of bias), and at review level (e.g., incomplete retrieval of identified research, reporting bias).	15-16
Conclusions	26	Provide a general interpretation of the results in the context of other evidence, and implications for future research.	16-17
FUNDING			
Funding	27	Describe sources of funding for the systematic review and other support (e.g., supply of data); role of funders for the systematic review.	2, 18

Material S2 Exemplary search strategies

PubMed

URL: https://www.ncbi.nlm.nih.gov/pubmed/advanced

Date: 17.01.19

Keywords:

(((((((((("pregnancy") OR "pregnant") OR "prenatal") OR "antenatal") OR "gestation") OR "gestational")) AND ((("intervention") OR "trial") OR "study")) AND (((((((((((((((((("exercise") OR "exercising") OR "physical activity") OR "diet") OR "dieting") OR "dietary") OR "nutrition") OR "nutritional") OR "lifestyle") OR "behavior") OR "behavioral") OR "behaviour") OR "behavioural") OR "counsel") OR "counseling") OR "counselling") OR "monitor") OR "monitoring") OR "advice") OR "advise")) AND ((((((((("infant") OR "infancy") OR "child") OR "children") OR "childhood") OR "offspring") OR "adolescence") OR "adolescent") OR "toddler") OR "youth")) AND ((((((((((((((((((("weight") OR "bmi") OR "body mass index") OR "percent") OR "percentile") OR "z score") OR "overweight") OR "obesity") OR "obese") OR "adiposity") OR "adipose") OR "anthropometry") OR "anthropometric") OR "metabolism") OR "metabolic") OR "circumference") OR "body fat") OR "fat mass") OR "lean mass") OR "body composition") OR "skinfold thickness")) AND ((((("random") OR "randomly") OR "randomized") OR "randomised") OR "rct")

Filters used: Humans only

Results before refining: 3589 references identified

Results after refining: 2931 references identified

Embase

URL: https://www.embase.com
Date: 17.01.2019
Keywords:
((pregnan* or "prenatal" or "antenatal" or gestation*) and (interven* or "trial" or "study") and (exercis* or "physical activity" or diet* or nutrition* or "lifestyle" or behavio* or counsel* or monitor* or "advice" or "advise") and (infan* or child* or "offspring" or adolescen* or "toddler" or "youth") and ("weight" or "bmi" or "body mass index" or percent* or "z score" or "overweight" or obes* or adipos* or anthropometr* or metabol* or "circumference" or "body fat" or "fat mass" or "lean mass" or "body composition" or "skinfold thickness") and (random* or "rct")).af.

Filters used: Humans only

Results before refining: 5371 references identified

Results after refining: 4792 references identified

Table S1 Detailed summary of study, maternal and infant characteristics

Study	Design, country, setting	Number of participants randomized and analyzed (%)	Length of follow-up	Maternal characteristics[b]	Infant characteristics[b]
**Chiavaroli et al. [1], 2018**	Design: Single-center RCT<br>Country: New Zealand<br>Setting: Community-based	N randomized: 98 (I: 49, C: 49)<br>N analyzed 7 y:<br>I: 33 (67%)<br>C: 24 (49%)	1 y, 7 y	GW: < 20<br>BMI class: All<br>Baseline BMI (kg/m^2):<br>I: 25.5 (4.3), C: 25.4 (2.9)<br>GWG (kg): N/R<br>Other: Only nulliparous women aged 20-40, who were relatively sedentary included	BW (g): I: 3426 (427), C: 3569 (433)<br>LGA: N/R<br>SGA: I: 4 (9%), C: 3 (8%)<br>BF: N/R
**Delta Healthy Sprouts [2]**	Design: RCT<br>Country: USA<br>Setting: Home visits	N randomized: 105 (I: 54, C: 51)<br>N analyzed 12 m:<br>I: 21 (39%)<br>C: 25 (49%)	Monthly 1-12 m	GW: < 19<br>BMI class: All<br>Baseline BMI (kg/m^2): N/R<br>GWG (kg): N/R<br>Other: Large majority of women were of African-American race and unmarried, from region with high rates of OB, diabetes, hypertension	BW (g): I: 3242 (476), C: 2994 (647)<br>LGA: N/R<br>SGA: N/R<br>BF: Majority of infants were fed formula within first 24h and half or less were ever breastfed<br>I: 12 (50%), C: 9 (30%)<br>Other: Sign. more females in I at birth (I: 14 (58%), C: 9 (30%), $P = 0.036$)
**ETIP [3, 4]**	Design: Single-center RCT<br>Country: Norway<br>Setting: University hospital	N randomized: 91 (I: 46, C: 45)<br>N analyzed 3 m:<br>I: 36 (78%)<br>C: 34 (76%)	6 wks, 3 m	GW: ≤ 18<br>BMI class: ≥ 28 kg/m^2<br>Baseline BMI (kg/m^2):<br>I: 33.9 (3.8), C: 35.2 (4.5)<br>GWG (kg): I: 10.5 (4.9), C: 9.2 (7.3)<br>Other: Only previously sedentary women included	BW (g): I: 3719 (699), C: 3912 (413)<br>LGA: I: 13 (35%), C: 19 (53%)<br>SGA: I: 2 (5%), C: 0 (0%)<br>BF: Excl. BF: I: 18 (60%), C: 21 (78%)

Table S1 (continued)

Study	Design, country, setting	Number of participants randomized and analyzed (%)	Length of follow-up	Maternal characteristics[b]	Infant characteristics[b]
**ETOIG [5]**	Design: Multi-center RCT<br>Country: France<br>Setting: University hospitals	N randomized: 275 (I: 138, C: 137)<br>N available 2 y:<br>I: 102 (74%)<br>C: 136 (76%)	6 m[a], 2 y[a]	GW: ≤ 21<br>BMI class: OW or OB<br>Baseline BMI (kg/m^2):<br>I: 32.5 (5.4), C: 32.5 (5.4)<br>GWG (kg): N/R<br>Other: Majority of women were found to be of high socioeconomic status and have high levels of education	BW (g) as mean (minimum-maximum):<br>I: 3420 (800–4730), C: 3420 (820–4850)<br>LGA: I: 24 (19%), C: 16 (12%)<br>SGA: I: 6 (5%), C: 11 (8%)<br>BF: Excl. BF at 4 m:<br>I: 27 (23%), C: 22 (18%)
**FeLIPO [6]**	Design: Multi-center, cluster-RCT<br>Country: Germany<br>Setting: Gynecological practices	N randomized: 250 individuals, 8 practices<br>(I: 4 practices, 167 participants,<br>C: 4 practices, 83 participants)<br>N analyzed 10-12 m:<br>I: 150 (90%)<br>C: 70 (84%)	1-2, 3-4, 6-7, 10-12 m	GW: < 18<br>BMI class: All, except underweight<br>Baseline BMI (kg/m^2) as median (IQR):<br>I: 22.2 (20.7 - 24.3), C: 23.3 (21.2 - 26.8), $P = 0.008$<br>GWG (kg): I: 14.1 (4.1), C: 15.6 (5.8)<br>Other: Majority of women were normal weight, German-born	BW (g): I: 3406 (402), C: 3414 (445)<br>LGA: I: 10 (6%), C: 7 (9%)<br>SGA: I: 6 (4%), C: 3 (4%)<br>BF: Excl. BF: I: 131 days, C: 116 days; Total BF: I: 232 days, C: 219 days<br>Other: N/R
**Fit for Delivery [7]**	Design: Single-center RCT<br>Country: USA<br>Setting: Research center	N randomized: 401 (I: 201, C: 200)<br>N analyzed 6 m:<br>I: 115 (57%)<br>C: 107 (54%)	4 m, 6 m	GW: 10-16<br>BMI class: Between 19.8 and 40 kg/m^2<br>Baseline BMI (kg/m^2): N/R<br>GWG (kg): I: 15.0 (5.7), C: 15.7 (6.2)<br>Other: Only non-smokers included, most were non-Hispanic White	BW (g): C: 3370 (560), I: 3390 (560)<br>LGA: N/R<br>SGA: N/R<br>BF: N/R<br>Other: Outcome data reported for NW vs. OW/OB women

Table S1 (continued)

Study	Design, country, setting	Number of participants randomized and analyzed (%)	Length of follow-up	Maternal characteristics[b]	Infant characteristics[b]
**GeliS [8]**	Design: Multi-center, cluster-RCT<br>Country: Germany<br>Setting: Public health, Gynecological and midwifery practices	N randomized: 2286 individuals, 10 clusters<br>(I: 5 cluster of 1139 participants, C: 5 clusters of 1122 participants)<br>N analyzed 10-12 m: I: 866 (76%)<br>C: 850 (76%)	1-2, 3-4, 6-7, 10-12 m	GW: < 12<br>BMI class: All, except underweight<br>Baseline BMI (kg/m^2): I: 24.4 (4.4), C: 24.3 (4.6)<br>GWG (kg): I: 14.1 (5.3), C: 14.1 (5.2)<br>Other: Women were predominantly White, relatively well educated, more nulliparous women in intervention group	BW (g): I: 3313 (536), C: 3363 (498)<br>LGA: I: 73 (7%), C: 75 (8%)<br>SGA: I: 88 (9%), C: 84 (8%)<br>BF: Excl. BF: I: 588 (87%), C: 558 (84%), $P = 0.019$
**HAPPY [9]**	Design: Single-center RCT<br>Country: UK<br>Setting: Community locations (e.g. children's centers)	N randomized: 120 (I: 59, C: 61)<br>N analyzed 12 m:<br>I: 38 (64%)<br>C: 40 (66%)	6 m, 12 m	GW: 10-26<br>BMI class: OW or OB<br>Baseline BMI (kg/m^2):<br>I: 29.5 (4.7), C: 30.0 (4.9)<br>GWG (kg): N/R<br>Other: Women were mostly of South Asian origin, city characterized by high levels of socioeconomic deprivation and ethnic diversity	BW (g): N/R<br>LGA: N/R<br>SGA: N/R<br>BF: N/R
**Healthy Mom Zone [10, 11]**	Design: Single-center RCT<br>Country: USA<br>Setting: Visit to research center and at-home participation	N randomized: 31 (I: 15, C: 16)<br>N analyzed 5-11 wks:<br>I: 9 (60%)<br>C: 10 (63%)	5-11 wks	GW: > 8<br>BMI class: OW or OB (25 to 45 kg/m^2)<br>Baseline BMI (kg/m^2):<br>I: 30.7 (6.7), C: 32.4 (7.5)<br>GWG (kg): N/R<br>Other: Most women were married, middle to upper class, Caucasian, from rural/ suburban areas	BW (g): I: 3379 (629), C: 3383 (629)<br>LGA: N/R<br>SGA: I: 2 (15%), C: 2 (14%)<br>BF: N/R<br>Other: Macrosomia: I: 2 (15%), C: 1 (7%)

Table S1 (continued)

**Study**	**Design, country, setting**	**Number of participants randomized and analyzed (%)**	**Length of follow-up**	**Maternal characteristics[b]**	**Infant characteristics[b]**
**Healthy Moms [12]**	Design: Single-center RCT<br>Country: USA<br>Setting: Closed-panel managed care organization	N randomized: 118 (I: 56, C: 58)<br>N analyzed 12 m:<br>I: 51 (91%)<br>C: 52 (90%)	12 m	GW: ≤ 20<br>BMI class: OB<br>Baseline BMI (kg/m^2):<br>I: 36.7 (5.2), C: 36.8 (4.7)<br>GWG (kg): I: 5.0 (4.1), C: 8.4 (4.7)<br>Other: Women were primarily White with at least a high school education. Over half were classified with class 2 or 3 obesity	BW (g): I: 3480 (480), C: 3680 (670)<br>LGA: N/R<br>SGA: N/R<br>BF: N/R
**Kong et al. [13] 2014**	Design: Single-center RCT<br>Country: USA<br>Setting: University	N randomized: 42 (I: 19, C: 23)<br>N analyzed 6 m:<br>I: 15 (79%)<br>C: 18 (78%)	1 m, 6 m	GW: < 15 GW<br>BMI class: OW or OB<br>Baseline BMI (kg/m^2):<br>I: 30.6 (5.1), C: 30.6 (4.1)<br>GWG (kg): I: 11.3 (7.2), C: 11.3 (7.4)<br>Other: Only non-exercising non-smokers included, cohort predominantly White, married, educated	BW (g): I: 3650 (474), C: 3774 (486)<br>LGA: N/R<br>SGA: N/R<br>BF: N/R<br>Other: Macrosomia: I: 5 (28%), C: 6 (32%)
**LIMIT [14-16]**	Design: Multi-center RCT<br>Country: Australia<br>Setting: Three public maternity hospitals	N randomized: 2212<br>(I: 1108, C: 1104)<br>N analyzed 3-5 y:<br>I: 727 (66%)<br>C: 691 (63%)	6 m,<br>18 m,<br>3-5 y	GW: 10-20 GW<br>BMI class: OW or OB<br>Baseline BMI (kg/m^2):<br>I: 31.6 (1.3), C: 31.3 (1.3)<br>GWG (kg): I: 9.4 (5.7), C: 9.4 (5.8)<br>Other: Women were predominantly White	BW (g): I: 3481 (554), C: 3492 (613)<br>LGA: I: 203 (19%), C: 224 (21%)<br>SGA: N/R<br>BF: Still being breastfed at 6 m old: I: 324 (59%), C: 347 (60%)<br>Mean duration of BF (m): I: 8.28, C: 8.69

Table S1 (continued)

Study	Design, country, setting	Number of participants randomized and analyzed (%)	Length of follow-up	Maternal characteristics[b]	Infant characteristics[b]
**LiPO [17]**	Design: Multi-center RCT<br>Country: Denmark<br>Setting: Two university hospitals	N randomized: 360 (I:180, C: 180)<br>N analyzed 2.8 y:<br>I: 82 (46%)<br>C: 75 (42%)	2.8 y	GW: 10-14 GW<br>BMI class: OB<br>Baseline BMI (kg/m^2):<br>I: 34.1 (3.2) C: 34.3 (3.1)<br>GWG (kg) as mean (95% CI): I: 7.7 (6.8 to 8.7), C: 8.8 (7.7 to 9.8)<br>Other: Women were exclusively Caucasian	BW (g): I: 3634 (714), C: 3616 (490)<br>LGA: I: 11 (13%), C: 8 (11%)<br>SGA: N/R<br>BF: Any BF at 6 m: I: 68 (55%), C: 62 (54%), full BF at 6 m: I: 29 (24%), C: 30 (26%)<br>Other: Macrosomia: I: 23 (29%); C: 16 (21%)
**NAMI [18]**	Design: Multi-center RCT, 4-arm study<br>Country: Finland<br>Setting: Municipal well-women clinics	N randomized: 171 (I: 86, C: 85)<br>N analyzed 6 m:<br>I: 73 (85%)<br>C: 70 (82%)	1 m, 6 m, 1 y[a], 2 y[a], 4 y[a]	GW: < 17 GW<br>BMI class: All<br>Baseline BMI (kg/m^2):<br>I: 24.3 (4.4), C: 23.7 (3.5)<br>GWG (kg): I: 14.8 (5.1), C: 14.9 (5.2)<br>Other: Women were exclusively White, majority had high education levels and were primiparous	BW (g): I: 3628 (385), C: 3600 (525)<br>LGA: N/R<br>SGA: N/R<br>BF: Excl. BF duration (m): I: 3.4, C: 3.0,<br>any BF at 6 m: I: 57 (75%), C: 53 (76%)<br>Other: N/R
**NELLI [19]**	Design: Multi-center, cluster-RCT<br>Country: Finland<br>Setting: Maternity clinics	N randomized: 442 individuals, 14 clusters<br>(I: 7 clusters of 246 participants<br>C: 7 clusters of 196 participants)<br>N analyzed 7 y:<br>I: 77 (31%)<br>C: 73 (37%)	7 y	GW: 8-12 GW<br>BMI class: All<br>Baseline BMI (kg/m^2):<br>I: 26.3 (4.9), C: 26.4 (4.3)<br>GWG (kg): I: 13.8 (5.8), C: 14.2 (5.1)<br>Other: Minimum 1 out of 4 risk factors required: BMI ≥ 25 kg/m^2, history of GDM/ macrosomic birth, age > 40 y, diabetes in family	BW (g): I: 3532 (514), C: 3659 (455) (*P* = 0.008)<br>LGA: I: 26 (12%), C: 34 (20%) (*P* = 0.042)<br>SGA: I: 10 (5%), C: 5 (3%)<br>BF: N/R<br>Other: Macrosomia: I: 37 (17%), C: 36 (21%)

Table S1 (continued)

Study	Design, country, setting	Number of participants randomized and analyzed (%)	Length of follow-up	Maternal characteristics[b]	Infant characteristics[b]
**RADIEL [20]**	Design: Multi-center RCT<br>Country: Finland<br>Setting: Maternity and general hospitals	N randomized: 728 (I:370, C: 358)<br>N analyzed 5 y:<br>I: 171 (46%),<br>C: 149 (42%)	6 m,<br>12 m, 5 y	GW: <20 GW, or planning pregnancy<br>BMI class: All<br>Baseline BMI (kg/m^2): Total: 32.2 (5.8)<br>GWG (kg): N/R<br>Other: History of GDM or BMI ≥ 30 kg/m^2 were required, women were exclusively White, majority well educated, non-smokers, primiparous	BW (g): I: 3640 (597), C: 3706 (524)<br>LGA: I: 8 (2%), C: 13 (6%)<br>SGA: N/R<br>BF: Excl. BF at 6 wks: I: 63%, C: 57%. Partial BF at 1 y: I: 32%, C: 26%
**ROLO [21, 22]**	Design: Single-center RCT<br>Country: Ireland<br>Setting: National maternity hospital	N randomized: 800 (I: 394, C: 406)<br>N analyzed 6 m:<br>I: 138 (35%)<br>C: 142 (35%)	3 m, 6 m, 2 y[a], 5 y[a]	GW: ≤ 18 GW<br>BMI class: All<br>Baseline BMI (kg/m^2):<br>I: 26.4 (4.7), C: 26.2 (4.6)<br>GWG (kg): I: 13.3 (4.5), C: 13.8 (4.9)<br>Other: Only secundigravid women with macrosomic birth included, majority were White	BW (g): I: 4100 (500), C: 4000 (500) ($P = 0.762$)<br>LGA: N/R<br>SGA: N/R<br>BF: Duration of BF (wks) I: 4.25, C: 3.3<br>Other:<br>Macrosomia: I: 189 (51%), C: 199 (51%)
**Stafne et al. [23], 2012**	Design: Multi-center RCT<br>Country: Norway<br>Setting: One hospital and two university hospitals	N randomized: 855 (I: 429, C: 426)<br>N analyzed 15 m:<br>I: 144 (34%)<br>C: 114 (27%)	6 wks, 3 m, 6 m, 9-10 m,<br>12 m, 15 m	GW: 18-22 GW<br>BMI class: All<br>Baseline BMI (kg/m^2):<br>I: 24.7 (3.0), C: 25.0 (3.4)<br>GWG (kg): N/R<br>Other: Only White women included, cohort generally exercised regularly, BMI mostly in normal range	BW (g): I: 3515 (534), C: 3523 (546)<br>LGA: N/R<br>SGA: N/R<br>BF: N/R<br>Other: Macrosomia: I: 71 (17%), C: 78 (18%)

Table S1 (continued)

Study	Design, country, setting	Number of participants randomized and analyzed (%)	Length of follow-up	Maternal characteristics[b]	Infant characteristics[b]
**UPBEAT [24]**	Design: Multi-center RCT<br>Country: United Kingdom<br>Setting: Hospitals	N randomized: 1555<br>(I: 783, C: 772)<br>N analyzed 6 m:<br>I: 332 (42%)<br>C: 345 (45%)	6 m	GW: 15-18 GW<br>BMI class: OB<br>Baseline BMI (kg/m^2):<br>I: 36.2 (5.0), C: 36.3 (4.7)<br>GWG (kg): I: 6.9 (4.7), C: 7.8 (4.4)<br>Other: Sample characterized by high ethnic diversity and socioeconomic deprivation; women aged ≥16 y were included	BW (g): I: 3479 (529), C: 3437 (604)<br>LGA: I: 30 (9%), C: 27 (8%)<br>SGA: N/R<br>BF: At 72 h pp: I: 63%, C: 61%. Excl. BF at 6 m: I: 3%, C: 3%, Duration excl. BF (days) I: 65%, C: 66%
**VIGA [25]**	Design: Multi-center RCT<br>Country: Sweden<br>Setting: Antenatal clinics	N randomized: 445<br>(I: 221, C: 224)<br>N analyzed 6 y:<br>I: 103 (47%)<br>C: 82 (37%)	Bi-monthly from birth to 12 m, at 18 m, and 2.5 y, 4 y, 5 y and 6 y	GW: ≤ 16 GW<br>BMI class: All except underweight<br>Baseline BMI (kg/m^2):<br>I: 25.2 (4.9), C: 25.3 (4.8)<br>GWG (kg): I: 14.2 (4.5), C: 15.3 (5.4)<br>Other: Larger proportion of normal weight women compared to the country population	BW (g): I: 3661 (461), C: 3548 (489)<br>LGA: I: 15 (8%), C: 11 (6%)<br>SGA: I: 3 (2%), C: 3 (2%)<br>BF: I: 60%, C: 62%<br>Other: Macrosomia: I: 47 (24%), C: 28 (15%)

Abbreviations: BF, breastfeeding; BMI, body mass index; BW, birth weight; CI, confidence interval; C, control; GW, gestational week; IQR, interquartile range; I, intervention; LGA, large for gestational age; m, months; N/R, not applicable; N/R, not reported; NW, normal weight; OB, obese; OW, overweight; RCT, randomized controlled trial; SGA, small for gestational age; wk, week; y, year

[a]Follow-ups not included in meta-analysis

[b]Data reported as mean (standard deviation) or N (%) unless otherwise indicated

Table S2 Intervention characteristics of included studies

Study	Duration	Theory & content	Setting & delivery	Timing & frequency	Adherence & fidelity
**Chiavaroli et al. [1], 2018**	Pregnancy (~20-36 GW)	Theory: N/R<br>Aim: Reduce offspring birth size and percentage of body fat, and attenuate reduction in maternal insulin sensitivity throughout pregnancy<br>Content: PA only: Aerobic exercise (stationary bicycles)<br>Materials: Stationary bicycles, training diary	Setting: Community-based: Home-based exercise program<br>Delivery: Individual-based, face-to-face<br>Provider: N/R	Fortnightly for ~ 15 wks (~ 7-8 contacts). Maximum of 5 sessions of 40 min exercise /wk, Familiarization (20 to 27 GW), maintenance period (GW 28 to 35)	Adherence: 75% of total exercise prescribed was performed during the full intervention period
**Delta Healthy Sprouts [2]**	Pregnancy and pp (~16 GW to 12 m pp)	Theory: Social cognitive theory and transtheoretical models of behavior change, DPP principles (flexible, culturally sensitive, individualized educational curriculum), elements of InFANT (anticipatory guidance theory, parenting support theory). Intervention targeted at women of African American race.<br>Aim: Improve infant growth outcomes (prevent childhood obesity), maternal GWG, optimize health behaviors<br>Content: Mixed: Diet, PA, weight management, infant feeding practices and other infant health behaviors<br>Materials: Eating plan, tracking sheets, GWG chart, DVDs	Setting: Community care: Home visits<br>Delivery: Individual-based, face-to-face, text-messaging<br>Provider: Trained Parent Educators (African American, college educated women residing in target communities)	Monthly over 18 m ~18 visits (90-120 min)	Adherence:<br>Pregnancy: Minimum 5 out of 6 visits: I: 51%, C: 79%<br>Pp: Mean number of postnatal visits (out of 12): I: 9.9, C: 10.2

Table S2 (continued)

**Study**	**Duration**	**Theory & content**	**Setting & delivery**	**Timing & frequency**	**Adherence & fidelity**
**ETIP [3, 4]**	Pregnancy (14 GW to delivery)	Theory: Motivational interviewing<br>Aim: Reduce GWG<br>Content: PA only: Supervised endurance and strength training and unsupervised training. Women advised to increase everyday PA.<br>Materials: Training diary, individualized GWG curve	Setting: Primary: Hospital<br>Delivery: Individual or group- based, face-to-face<br>Provider: Physical therapist (PA sessions)	Weekly: 2-4 supervised training sessions (60 min) + 1 at home training session (50 min) + 1 motivational interview (in each trimester) (30 min)	Adherence:<br>Mean sessions/ wk: 1.3 supervised, 0.8 unsupervised<br>56% exercised per protocol
**ETOIG [5]**	Pregnancy and pp (21 GW to 2 m pp)	Theory: Group therapeutic education<br>Aim: Reduce postnatal excessive weight gain in infants<br>Content: Mixed: Advice on diet, PA, healthy GWG, infant feeding and overall health. PA performed at the end of each session. Individually tailored education plan developed.<br>Materials: Education plan, national nutrition booklet	Setting: Primary: University hospitals<br>Delivery: Individual and group-based sessions, face-to- face<br>Provider: Physician (pediatrician or endocrinologist), dietician, midwife	Minimum 2 individual sessions (at 26 and 30 GW) + 4 group sessions (at 21, 28, 35 GW and 2 m pp)	Adherence:<br>Attendance of group sessions:<br>1st: 87%, 2nd: 77%, 3rd: 67%, 4th: 62%;<br>Attendance of individual session:<br>1st: 83%, 2nd: 72%;<br>63% followed entire program;<br>71% attended minimum 1 individual and 3 group sessions
**FeLIPO [6]**	Pregnancy (20 to 30 GW)	Theory: N/R<br>Aim: Prevent GWG in excess of IOM criteria<br>Content: Mixed: Diet, PA, GWG monitoring. Personalized feedback on health behaviors provided. Self-monitoring, behavior goals.<br>Materials: Weight gain chart	Setting: Primary<br>Delivery: Face-to-face<br>Provider: Trained researchers	2 counselling sessions (1st: 60 min, 2nd: 30 min)	Adherence:<br>93% attended both counselling sessions

Table S2 (continued)

Study	Duration	Theory & content	Setting & delivery	Timing & frequency	Adherence & fidelity
**Fit for Delivery [7]**	Pregnancy	Theory: Social learning theory (behavioral principles)<br>Aim: Reduce excessive GWG and promote return to pre-pregnancy weight by 12m pp<br>Content: Mixed: Personalized advice on diet PA, and emphasis on GWG monitoring. If GWG guidelines are not met additional phone calls and meal plans provided.<br>Materials: Body-weight scales, food records, pedometers, personalized GWG graph	Setting: Primary (research center)<br>Delivery: Individual face-to-face sessions, phone calls, automated postcards mailed weekly<br>Provider: Dietician, Interventionist	1 in-person visit + 3 phone calls (10-15 min) + additional phone calls if GWG guidelines not met	N/R
**GeliS [8]**	Pregnancy and pp (< 12 GW to 6-8 wks pp)	Theory: N/R<br>Aim: Prevent GWG in excess of IOM recommendations and obstetric complications, reduce the risk of maternal and offspring obesity<br>Content: Mixed: Diet, PA, and GWG monitoring components, partially individualized. Information on postnatal exercise, breastfeeding, and infant feeding practices provided.<br>Materials: Brochures, list of local PA programs, pedometer, weight gain chart	Setting: Primary (Gynecological and midwifery practices, real-life setting of routine prenatal care)<br>Delivery: Individual, face-to-face<br>Provider: Midwives gynecologists, medical staff	4 sessions:<br>12-16, 16-20, and 30-34 GW, and 6-8 wks pp (30-45 min each)	Adherence:<br>In total, 88% attended all 4 appointments.<br>1st: 98%, 2nd: 98%, 3rd: 96%,<br>pp session: 94%<br>3% did not attend any counselling session.<br>Mean number of attended sessions: 3.7

Table S2 (continued)

Study	Duration	Theory & content	Setting & delivery	Timing & frequency	Adherence & fidelity
**HAPPY [9]**	Pregnancy and pp (26-28 GW to 9 m pp)	Theory: Behavioral theory based on determinants of health behaviors (knowledge, motivation, social norms, skills and competencies), standardized behavior change techniques<br>Aim: Reducing infant obesity<br>Content: Diet and PA of mother and infant, culturally adapted, breastfeeding and positive parenting skills were encouraged<br>Materials: Handouts, food diaries, leaflet about PA, quiz sheet	Setting: Community locations (e.g. children's centers)<br>Delivery: Group sessions<br>Provider: Parenting facilitators	12 group sessions (6 antenatal, 6 postnatal) sessions (~ 2.5 h each)	Adherence: Average attendance: 2.19 (2.99) antenatal and 1.41 (1.43) postnatal sessions.<br>Attendance of antenatal sessions: ≥ 1: 44%, ≥ 4: 36%, all 6: 24%<br>Attendance of postnatal sessions: ≥ 3: 31%, ≥ 4: 22%<br>Fidelity: Only minor content changes
**Healthy Mom Zone [10, 11]**	Pregnancy (~8-36 GW)	Theory: Theory of planned behavior and self-regulation. Key intervention components: education, goal-setting/action plans, self-monitoring, and active learning<br>Aim: Managing GWG<br>Content: Diet, PA: Individually tailored, adaptations (maximum 5) based on GWG<br>Materials: Customized PA and diet booklets, mHealth tools for self-monitoring: GWG plots, Wi-Fi body weight scale, food scale, food diaries, accelerometers	Setting: Primary (research center) and at-home participation<br>Delivery: Face-to-face, mHealth tools, emails<br>Provider: Study dietitian, fitness instructor	Weekly meetings from ~8-36 GW	N/R
**Healthy Moms [12]**	Pregnancy	Theory: Behavior change intervention techniques, motivational interviewing techniques<br>Content: Diet, PA, behavior change, goal-setting<br>Materials: weigh-ins, food diaries, activity logs, pedometers, sample meals	Delivery: Two individual sessions, weekly group sessions (7-8 women) thereafter<br>Provider: Dietician	Weekly sessions (90 min each)	Adherence: Attended sessions: 20 (range 0-28)

Table S2 (continued)

Study	Duration	Theory & content	Setting & delivery	Timing & frequency	Adherence & fidelity
**Kong et al. [13], 2014**	Pregnancy (< 15 to ≥ 35 GW - minimum 20 wks)	Theory: N/R<br>Aim: Increase moderate-intensity PA in women and improve pregnancy and birth outcomes<br>Content: PA only. Unsupervised walking program, 2wks acclimation with increasing intensity<br>Materials: Treadmills, PA logs, ankle. worn accelerometer	Setting: Primary care<br>Delivery: One face-to-face training session, unsupervised program thereafter<br>Provider: N/R	Recommendation to walk 150 min weekly (spread as e.g. 30 min./ day for 5 days/ wk)	Adherence: Participants compliant in wearing measurement tool
**LIMIT [14-16]**	Pregnancy (10-20 to 36 GW)	Theory: Tailoring of interventions was informed by stage theories of health decision making. Goal-setting, barrier identification, self-monitoring was encouraged<br>Aim: Limit GWG in women<br>Content: Diet and PA advice, behavioral strategies<br>Materials: Written dietary and PA information, individual diet and PA plan, diary, recipe book example menu plans	Setting: Primary care<br>Delivery: Face-to-face sessions and phone calls<br>Provider: Research dietician and trained research assistants	3 in person sessions at trial entry, 28 GW, 36 GW + 3 phone calls at 22, 24, 32 GW	Adherence: 87% attended their first dietary appointment and 77% the second.<br>Fidelity: Consistent intervention provision
**LiPO [17]**	Pregnancy (15 to 35 GW)	Theory: N/R<br>Aim: Limit GWG in women to 5 kg<br>Content: Individualized dietary counselling and PA initiation (aerobic classes, gym membership, motivational coaching)<br>Materials: Website, pedometer	Setting: Primary and community care<br>Delivery: Face-to-face<br>Provider: Trained dieticians, physiotherapists	Diet: 4 sessions at 15, 20, 28, 35 GW<br>PA: 4-6 coaching sessions + Weekly 1 h gym class + women encouraged to be active 30-60 min/ day and to use 6 m gym membership	Adherence: 92% of women completed all four diet sessions, 98% completed min. 3. 56% attended aerobic classes for at least half the lessons. 77.5% undertook further leisure time sporting activities.

Table S2 (continued)

Study	Duration	Theory & content	Setting & delivery	Timing & frequency	Adherence & fidelity
**NAMI [18]**	Pregnancy and pp (14 GW to 6 m pp)	Theory: N/R<br>Aim: Modify dietary fatty acid composition in conjunction with balanced diet (high fiber), and optimize offspring diet to improve child health<br>Content: Intensive dietary counselling + placebo intake + feedback and recommendations on offspring diet<br>Materials: Favorable foods provided	Setting: Primary<br>Delivery: Face-to-face<br>Provider: Nutritionist	4 sessions at 14, 24, 34 GW and 6 m pp	N/R
**NELLI [19]**	Pregnancy (First clinic visit ~ 8-12 GW to 37 GW)	Theory: PRECEDE-PROCEED and Stages of Change.<br>Aim: Optimize maternal diet, PA, GWG to prevent GDM<br>Content: PA and dietary counselling, meetings including group exercise<br>Materials: Weight gain chart, notebooks, take-home leaflet, weekly action plan	Setting: Primary and community care (PA meeting)<br>Delivery: Face-to-face, individual counselling, group exercise sessions<br>Provider: Nurses, trained instructors/ physiotherapists	2 primary sessions (one on PA and one on diet, 20 to 30 min each) + 7 booster sessions (10-15 min each) + monthly group meetings on PA including exercise (2 h each)	N/R
**RADIEL [20]**	Preconception/ early pregnancy to 12 m pp	Theory: N/R<br>Aim: Prevent GDM, limit GWG, and later T2D, and CVD risk factors, optimize infant nutrition and family lifestyle<br>Content: PA and dietary counselling ("the plate model"), free access to exercise groups or pools provided<br>Materials: Pedometer, PA logbooks	Setting: Primary care<br>Delivery: Individual and group sessions, face-to-face<br>Provider: Nutritionist, PA advisor, study nurse	Session at enrolment, every 3 m before and during pregnancy, 6 wks, 6 m and 12 m pp + women encouraged to exercise 30 min/ day on 5 days/ wk	N/R

Table S2 (continued)

Study	Duration	Theory & content	Setting & delivery	Timing & frequency	Adherence & fidelity
**ROLO [21, 22]**	Pregnancy (14-34 GW)	Theory: N/R<br>Aim: Reduce postprandial blood glucose peaks via low GI diet, thereby reducing incidence of recurrent macrosomia<br>Content: Low GI diet and general healthy advice<br>Materials: Low GI recipes, list of low GI foods, emails	Setting: Primary care<br>Delivery: Groups of 2-6 women, face-to-face<br>Provider: Research dietician	Single 1-2 h session at 14 GW, reinforcement sessions at 28 and 34 GW	N/R
**Stafne et al. [23], 2012**	Pregnancy (20 to 36 GW)	Theory: N/R<br>Aim: Prevent GDM and improve insulin resistance<br>Content: Supervised and unsupervised PA (aerobic, strength, balance)<br>Materials: Written home exercise program, training diary	Setting: Primary care and at home participation<br>Delivery: Group-based face-to-face sessions and unsupervised individual PA<br>Provider: Physiotherapists	Weekly 60 min for 12 weeks + 45 min unsupervised PA twice weekly	Adherence: 55% of women exercised ≥ 3 days/ week<br>Fidelity: N/R
**UPBEAT [24]**	Pregnancy (15-18 GW to end of intervention period)	Theory: Control theory with components of social cognitive theory. SMART goals set. Motivational interviewing and behavior change techniques (advice on approaching barriers; finding social support; and opportunities for social comparison)<br>Aim: Reduce risk of GDM, delivery of LGA infants, and long-term risk of offspring obesity<br>Content: Dietary advice for reducing glycemic load and saturated fat intake, increasing everyday PA<br>Materials: Pedometer, participant handbook, exercise DVD, logbook for recording personal goals	Setting: Primary care (antenatal clinic)<br>Delivery: Face-to-face, missed sessions covered via phone or email<br>Provider: Health trainer	8 sessions, 1-1.5 h on a weekly basis	Adherence: 54% of women attended ≥ 4 sessions in person, 83% of women received ≥ 4 sessions in person or via telephone/email

Table S2 (continued)

Study	Duration	Theory & content	Setting & delivery	Timing & frequency	Adherence & fidelity
**VIGA [25]**	Pregnancy and pp (≤ 16 GW to pp)	Theory: Motivational toolbox used<br>Aim: Limit excessive GWG, illicit long-term behavioral change<br>Content: Formal prescription of exercise, education on recommended GWG<br>Materials: Personalized weight gain graph	Setting: Primary care<br>Delivery: Face-to-face<br>Provider: Midwives	7-8 sessions integrated into standard antenatal care visits	Fidelity: No record of women not receiving allocated intervention in either treatment group.

Abbreviations: CVD, cardiovascular disease; DPP, Diabetes prevention programme; DVD, digital versatile disc; GDM, gestational diabetes mellitus; GI, glycemic index; GW, gestational week; GWG, gestational weight gain; h, hour; InFANT, Infant Feeding Activity and Nutrition Trial; LGA, large for gestational age; m, month; min, minute; N/R, not reported; PA, physical activity; pp, postpartum; T2D, type 2 diabetes; wk, week

Table and text S3 Summary of GRADE assessment results

Outcome	Number of studies	Design	Risk of bias within studies	Heterogeneity	Precision of effect estimate	Directness of evidence	Publication bias	Overall strength of evidence
**Weight**	19	Randomized controlled trial	High	12-66%	High	Moderate	Likely none	Moderate
**Length**	17	Randomized controlled trial	High	0-71%	High	Moderate	Very likely none	Moderate
**BMI**	12	Randomized controlled trial	High	0-44%	High	Moderate	Very likely none	Moderate

As only randomized controlled trials were included, the strength of the body of evidence was initially considered to be high in terms of the primary outcomes [26]. Downgrading based on the moderate directness of evidence was considered inappropriate, given the inherently variable nature of lifestyle interventions and the explorative objective of this review. However, due to the high risk of bias observed in a considerable proportion of studies contributing data to weight, length, and BMI analyses, the rating was downgraded to moderate for all three outcomes. The 95% confidence intervals obtained through meta-analysis suggested high precision of the effect estimate for all three primary outcomes. The I^2 statistic indicated moderate to substantial heterogeneity. However, given the magnitude and direction of effects of individual studies, and that confidence intervals of the I^2 statistic suggested very low precision, it was not considered appropriate to further downgrade the level of evidence [26]. In terms of publication bias, neither visual inspection nor Egger's test indicated funnel plot asymmetry for 17 out of 18 age ranges (Figure S1). For the outcome weight, one age range (over 12 month to under 3 year old children) including data from four studies showed funnel plot asymmetry (Egger's test p=0.045). Based on these criteria, the strength of the body of evidence was considered to be moderate in terms of the outcomes weight, length, and BMI.

Table S4 Multivariate random-effects meta-analyses of weight, length, and BMI

Age	Weight (kg) $I^2 = 37\%$		Length (cm) $I^2=16\%$		BMI (kg/m²) $I^2=12\%$	
	Mean difference	95% CI	Mean difference	95% CI	Mean difference	95% CI
**1 to 2 months old**	−0.01	−0.10 to 0.08	0.06	−0.26 to 0.37	−0.05	−0.26 to 0.16
**3 to 4 months old**	−0.03	−0.10 to 0.05	0.03	−0.24 to 0.30	0.02	−0.09 to 0.14
**5 to 6 months old**	0.02	−0.06 to 0.10	0.20	−0.04 to 0.44	0.03	−0.07 to 0.12
**Over 6 to 12 months old**	0.00	−0.11 to 0.12	0.17	−0.02 to 0.36	0.02	−0.10 to 0.14
**Over 12 months to under 3 years old**	0.04	−0.08 to 0.16	−0.03	−0.30 to 0.23	0.14*	0.02 to 0.26
**3 years and older**	−0.09	−0.40 to 0.23	−0.23	−0.79 to 0.33	0.00	−0.21 to 0.21

Abbreviations: CI, confidence interval
*statistically significant effect estimate ($p < 0.05$)

Table S5 Summary of subgroup analyses

Table S5 (A) Summary of subgroup analyses of the association of prenatal interventions with child weight (kg)

	Time range	N studies	N intervention	N control	Mean difference (kg)	95% CI	I^2 (%)
**High-risk population**	1	3	50	55	− 0.19	−0.45 - 0.07	0%
	2	2	247	245	− 0.10	−0.66 - 0.46	47%
	3	6	1856	1860	0.00	−0.09 - 0.10	18%
	4	3	354	353	−0.02	−0.19 - 0.16	0%
	5	2	1153	1140	0.03	−0.22 - 0.28	28%
	6	3	1313	1278	−0.10	−0.64 - 0.44	53%
**General population**	1	5	1365	1215	0.02	−0.13 - 0.17	80%
	2	6	1493	1313	0.00	−0.12 - 0.13	60%
	3	7	1557	1394	0.03	−0.10 - 0.16	53%
	4	6	1419	1249	0.07	−0.13 - 0.26	66%
	5	2	297	251	0.24	0.01 - 0.47	0%
	6	2	136	106	−0.15	−0.97 - 0.67	0%
**Mixed interventions**	1	4	1052	964	−0.11	−0.16 - −0.05	0%
	2	4	1158	1048	−0.07	−0.14 - 0.00	0%
	3	8	2855	2753	−0.02	−0.08 - 0.04	0%
	4	6	1391	1298	−0.06	−0.23 - 0.10	49%
	5	2	1153	1140	0.03	−0.22 - 0.28	28%
	6	3	1313	1278	−0.10	−0.64 - 0.44	53%
**Physical activity interventions**	1	4	363	306	0.04	−0.14 - 0.22	57%
	2	3	371	299	0.07	−0.17 - 0.30	64%
	3	3	347	289	0.15	−0.11 - 0.40	59%
	4*	3	382	304	0.24	0.07 - 0.41	0%
	5	2	297	251	0.24	0.01 - 0.47	0%
	6	2	136	106	−0.15	−0.97 - 0.67	0%
**Dietary interventions**	1	NA	NA	NA	NA	NA	NA
	2	NA	NA	NA	NA	NA	NA
	3	2	211	212	0.12	−0.18 - 0.41	34%
	4	NA	NA	NA	NA	NA	NA
	5	NA	NA	NA	NA	NA	NA
	6	NA	NA	NA	NA	NA	NA

Table S5 (A) (continued)

	Time range	N studies	N intervention	N control	Mean difference (kg)	95% CI	I^2 (%)
**Interventions ending before childbirth**	1	5	371	252	−0.01	−0.12 - 0.10	0%
	2	5	693	548	−0.06	−0.19 - 0.06	9%
	3	7	1994	1880	−0.03	−0.12 - 0.05	8%
	4	4	411	268	−0.09	−0.38 - 0.21	62%
	5	3	1297	1254	0.05	−0.11 - 0.22	27%
	6	3	1175	1153	−0.42	−1.37 - 0.53	55%
**Interventions continuing postpartum**	1	3	1044	1018	0.02	−0.26 - 0.29	88%
	2	3	1047	1010	0.10	−0.16 - 0.37	79%
	3	6	1419	1374	0.10	−0.04 - 0.24	47%
	4	5	1362	1334	0.11	−0.02 - 0.24	28%
	5	NA	NA	NA	NA	NA	NA
	6	2	274	231	0.06	−0.48 - 0.60	0%

Abbreviations: CI, confidence interval
*Test for subgroup difference significant ($p < 0.05$)

Table S5 (B) Summary of subgroup analyses of the association of prenatal interventions with child length (cm)

	Time range	N studies	N intervention	N control	Mean difference (cm)	95% CI	I^2 (%)
**High-risk population**	1	2	27	29	−0.57	−1.83 - 0.69	0%
	2	NA	NA	NA	NA	NA	NA
	3	6	1821	1835	0.04	−0.20 - 0.29	0%
	4	3	343	342	−0.13	−0.57 - 0.31	0%
	5	2	1153	1140	−0.14	−0.49 - 0.20	0%
	6	3	1312	1279	−0.04	−0.51 - 0.42	0%
**General population**	1	5	1365	1213	0.23	−0.33 - 0.79	78%
	2	5	1373	1209	0.13	−0.36 - 0.61	71%
	3	6	1435	1287	0.23	−0.17 - 0.63	59%
	4	6	1418	1249	0.20	−0.06 - 0.45	11%
	5	2	297	251	0.30	−0.26 - 0.85	0%
	6	2	136	106	−0.58	−1.87 - 0.71	0%
**Mixed interventions**	1	4	1052	963	−0.20	−0.67 - 0.27	33%
	2*	3	1043	944	−0.28	−0.52 - −0.03	0%
	3	7	2704	2621	−0.06	−0.23 - 0.12	0%
	4	6	1379	1287	0.00	−0.22 - 0.22	0%
	5	2	1153	1140	−0.14	−0.49 - 0.20	0%
	6	3	1312	1279	−0.04	−0.51 - 0.42	0%
**Physical activity interventions**	1*	3	340	279	0.51	0.10 - 0.92	7%
	2*	2	330	265	0.48	0.10 - 0.86	0%
	3*	3	341	289	0.63	0.24 - 1.01	0%
	4*	3	382	304	0.49	0.09 - 0.88	0%
	5	2	297	251	0.30	−0.26 - 0.85	0%
	6	2	136	106	−0.58	−1.87 - 0.71	0%
**Dietary interventions**	1	NA	NA	NA	NA	NA	NA
	2	NA	NA	NA	NA	NA	NA
	3	2	211	212	0.24	−0.33 - 0.81	0%
	4	NA	NA	NA	NA	NA	NA
	5	NA	NA	NA	NA	NA	NA
	6	NA	NA	NA	NA	NA	NA

Table S5 (B) (continued)

	Time range	N studies	N intervention	N control	Mean difference (cm)	95% CI	I^2 (%)
**Interventions ending before childbirth**	1	4	348	226	0.09	−0.34 - 0.52	0%
	2	2	331	200	0.05	−0.53 - 0.63	43%
	3	6	1868	1766	0.12	−0.13 - 0.37	0%
	4	4	404	257	−0.01	−0.59 - 0.57	34%
	5	3	1297	1254	−0.07	−0.38 - 0.25	0%
	6	3	1175		−0.11	−0.63 - 0.40	0%
**Interventions continuing postpartum**	1	3	1044	1016	0.36	−0.65 - 1.36	88%
	2	3	1042	1009	0.29	−0.61 - 1.18	82%
	3	6	1388	1356	0.17	−0.24 - 0.58	54%
	4	5	1357	1334	0.14	−0.08 - 0.36	2%
	5	NA	NA	NA	NA	NA	NA
	6	2	273	231	−0.09	−0.92 - 0.75	0%

Abbreviations: CI, confidence interval
*Test for subgroup difference significant ($p < 0.05$)

Table S5 (C) Summary of subgroup analyses of the association of prenatal interventions with child BMI (kg/m^2)

	Time range	N studies	N intervention	N control	Mean difference (kg/m^2)	95% CI	I^2 (%)
**High-risk population**	1	NA	NA	NA	NA	NA	NA
	2	NA	NA	NA	NA	NA	NA
	3	3	1347	1337	0.04	−0.12- 0.19	8%
	4	2	303	301	0.04	−0.19 - 0.28	0%
	5	2	1153	1140	0.10	−0.03 - 0.24	0%
	6	3	1312	1278	−0.06	−0.40 - 0.28	67%
**General population**	1	5	1364	1204	−0.08	−0.31 - 0.16	53%
	2	5	1359	1208	0.01	−0.11 - 0.13	0%
	3	5	1360	1216	0.01	−0.17 - 0.19	31%
	4	6	1417	1248	0.03	−0.20 - 0.25	53%
	5	2	296	250	0.28	0.02 - 0.55	0%
	6	2	136	106	0.06	−0.32 - 0.44	0%
**Mixed interventions**	1*	4	1052	954	−0.22	−0.35 - −0.09	0%
	2	3	1030	944	−0.03	−0.17 - 0.11	0%
	3	6	2382	2283	0.00	−0.12 - 0.13	15%
	4	5	1339	1246	−0.06	−0.27 - 0.15	44%
	5	2	1153	1140	0.11	−0.04 - 0.26	7%
	6	3	1312	1278	− 0.06	−0.40 - 0.28	67%
**Physical activity interventions**	1	2	321	260	0.11	−0.18 - 0.41	20%
	2	2	329	264	0.12	−0.12 - 0.36	0%
	3	2	325	270	0.13	−0.11 - 0.38	7%
	4	3	381	303	0.22	0.00 - 0.43	0%
	5	2	296	250	0.28	0.02 - 0.55	0%
	6	2	136	106	0.06	−0.32 - 0.44	0%
**Dietary interventions**	1	NA	NA	NA	NA	NA	NA
	2	NA	NA	NA	NA	NA	NA
	3	NA	NA	NA	NA	NA	NA
	4	NA	NA	NA	NA	NA	NA
	5	NA	NA	NA	NA	NA	NA
	6	NA	NA	NA	NA	NA	NA

Table S5 (C) (continued)

	Time range	N studies	N intervention	N control	Mean difference (kg/m^2)	95% CI	I^2 (%)
**Interventions ending before childbirth**	1	3	330	199	−0.06	−0.36 - 0.23	0%
	2	2	318	200	−0.11	−0.40 - 0.17	7%
	3	3	1394	1268	−0.07	−0.26 - 0.11	27%
	4	3	360	216	−0.13	−0.61 - 0.34	70%
	5	3	1297	1254	0.11	−0.02 - 0.24	0%
	6	3	1175	1153	−0.15	−0.64 - 0.36	68%
**Interventions continuing postpartum**	1	3	1043	1015	−0.07	−0.50 - 0.35	76%
	2	3	1041	1008	0.04	−0.10 - 0.17	0%
	3	5	1313	1285	0.09	−0.03 - 0.21	0%
	4	5	1360	1333	0.08	− 0.03 - 0.20	0%
	5	NA	NA	NA	NA	NA	NA
	6	2	273	231	0.05	−0.22 - 0.32	0%

Abbreviations: CI, confidence interval
*Test for subgroup difference significant ($p < 0.05$)

Table S6 Summary of sensitivity analyses

Table S6 (A) Summary of sensitivity analyses of the association of prenatal interventions with child weight (kg)

	Time range	N studies	N intervention	N control	Mean difference (kg)	95% CI	I^2 (%)
**Excluding studies at a high risk of bias**	1	5	1064	1034	− 0.05	−0.27 - 0.18	83%
	2	4	1178	1122	− 0.01	−0.21 - 0.18	72%
	3	9	2934	2883	0.03	−0.06 - 0.11	43%
	4	5	1392	1361	0.08	−0.06 - 0.23	44%
	5	2	1224	1202	0.13	−0.26 - 0.52	72%
	6	3	1339	1287	0.06	−0.17 - 0.29	0%
**Excluding cluster-randomized controlled trials**	1	6	393	345	0.04	−0.11 - 0.19	35%
	2	6	717	638	0.06	−0.10 - 0.21	39%
	3	11	2394	2334	0.05	−0.04 - 0.15	38%
	4	7	757	682	0.11	−0.03 - 0.25	15%
	5	4	1450	1391	0.13	−0.07 - 0.33	46%
	6	4	1372	1311	0.05	−0.17 - 0.28	0%

Abbreviations: CI, confidence interval

Table S6 (B) Summary of sensitivity analyses of the association of prenatal interventions with child length (cm)

	Time range	N studies	N intervention	N control	Mean difference (cm)	95% CI	I^2 (%)
**Excluding studies at a high risk of bias**	1	3	1041	1006	0.08	−0.81 - 0.98	86%
	2	2	1022	984	0.15	−0.77 - 1.08	90%
	3	8	2777	2751	0.12	−0.16 - 0.41	44%
	4	5	1380	1350	0.10	−0.22 - 0.42	33%
	5	2	1224	1202	−0.09	−0.42 - 0.25	0%
	6	3	1338	1287	−0.05	−0.51 - 0.40	0%
**Excluding cluster-randomized controlled trials**	1	5	370	318	0.43	−0.07 - 0.93	24%
	2*	3	350	290	0.50	0.13 - 0.87	0%
	3	10	2238	2202	0.25	0.00 - 0.50	20%
	4	7	746	671	0.21	−0.13 - 0.56	15%
	5	4	1450	1391	−0.02	−0.31 - 0.27	0%
	6	4	1371	1311	−0.07	−0.52 - 0.38	0%

Abbreviations: CI, confidence interval
*statistically significant effect estimate ($p < 0.05$)

Table S6 (C) Summary of sensitivity analyses of the association of prenatal interventions with child BMI (kg/m^2)

	Time range	N studies	N intervention	N control	Mean difference (kg/m^2)	95% CI	I^2 (%)
**Excluding studies at a high risk of bias**	1	2	1022	987	−0.03	−0.48 - 0.42	87%
	2	2	1021	983	0.07	−0.14 - 0.29	40%
	3	5	2366	2324	0.05	−0.05 - 0.14	0%
	4	4	1339	1308	0.09	−0.03 - 0.20	0%
	5	2	1223	1201	0.16	−0.07 - 0.39	46%
	6	3	1338	1287	0.11	−0.02 - 0.24	0%
**Excluding cluster-randomized controlled trials**	1	4	351	298	0.11	−0.13 - 0.36	0%
	2	3	349	289	0.12	−0.12 - 0.36	0%
	3	6	1690	1633	0.05	−0.06 - 0.17	0%
	4	6	705	629	0.13	−0.03 - 0.29	0%
	5*	4	1449	1390	0.14	0.02 - 0.26	0%
	6	4	1371	1311	0.10	−0.03 - 0.24	0%

Abbreviations: CI, confidence interval
*statistically significant effect estimate ($p < 0.05$)

Fig.S1 Funnel plots of (A-F) weight, (G-L) length, and (M-R) BMI data for the six defined age ranges

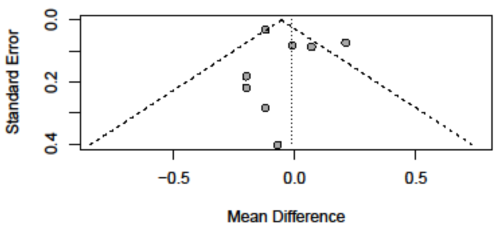

Fig. S1 (A) Funnel plot of weight data in 1 to 2 month old children (Egger's test for funnel plot asymmetry: p = 0.597)

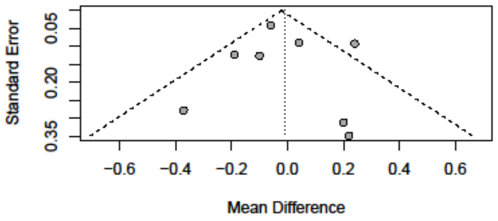

Fig. S1 (B) Funnel plot of weight data in 3 to 4 month old children (Egger's test for funnel plot asymmetry: p = 0.769)

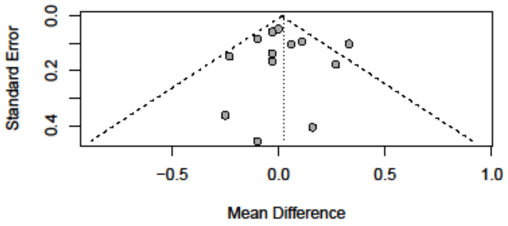

Fig. S1 (C) Funnel plot of weight data in 5 to 6 month old children (Egger's test for funnel plot asymmetry: p = 0.800)

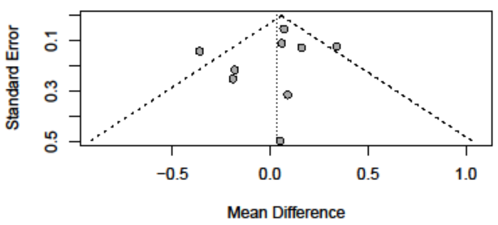

Fig. S1 (D) Funnel plot of weight data in over 6 to 12 month old children (Egger's test for funnel plot asymmetry: p = 0.540)

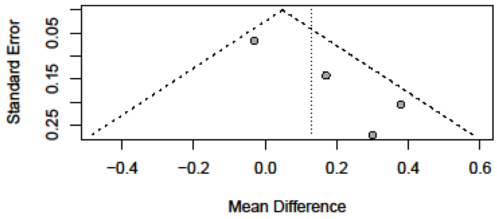

Fig. S1 (E) Funnel plot of weight data in over 12 month to under 3 year old children (Egger's test for funnel plot asymmetry: p = 0.045)

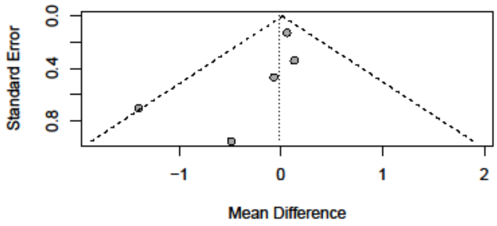

Fig. S1 (F) Funnel plot of weight data in 3 year and older children (Egger's test for funnel plot asymmetry: p = 0.211)

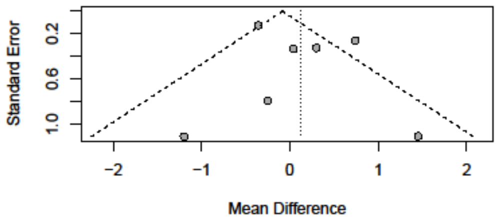

Fig. S1 (G) Funnel plot of length data in 1 to 2 month old children (Egger's test for funnel plot asymmetry: p = 0.390)

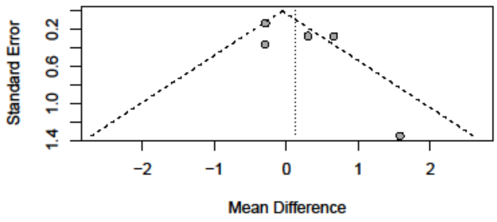

Fig. S1 (H) Funnel plot of length data in 3 to 4 month old children (Egger's test for funnel plot asymmetry: p = 0.254)

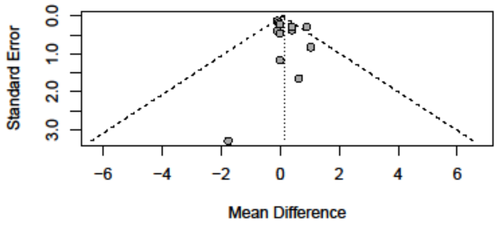

Fig. S1 (I) Funnel plot of length data in 5 to 6 month old children (Egger's test for funnel plot asymmetry: p = 0.272)

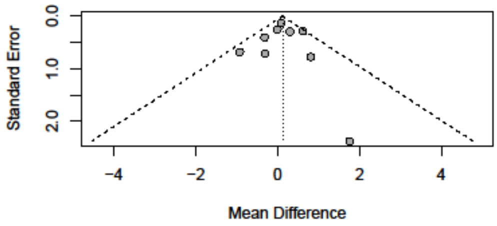

Fig. S1 (J) Funnel plot of length data in over 6 to 12 month old children (Egger's test for funnel plot asymmetry: p = 0.992)

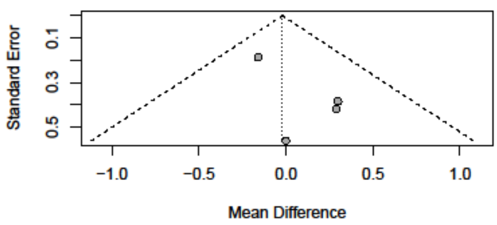

Fig. S1 (K) Funnel plot of length data in over 12 month to under 3 year old children (Egger's test for funnel plot asymmetry: p = 0.222)

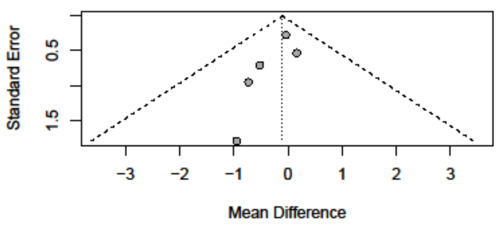

Fig. S1 (L) Funnel plot of length data in 3 year and older children (Egger's test for funnel plot asymmetry: p = 0.158)

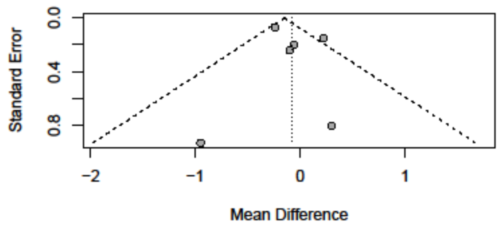

Fig. S1 (M) Funnel plot of BMI data in 1 to 2 month old children (Egger's test for funnel plot asymmetry: p = 0.535)

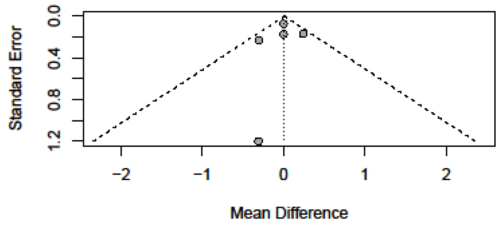

Fig. S1 (N) Funnel plot of BMI data in 3 to 4 month old children (Egger's test for funnel plot asymmetry: p = 0.778)

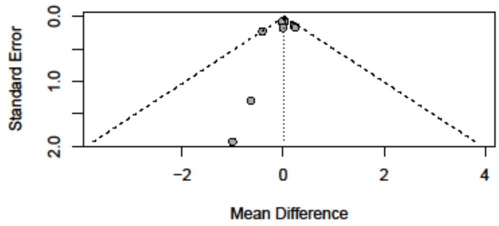

Fig. S1 (O) Funnel plot of BMI data in 5 to 6 month old children (Egger's test for funnel plot asymmetry: p = 0.591)

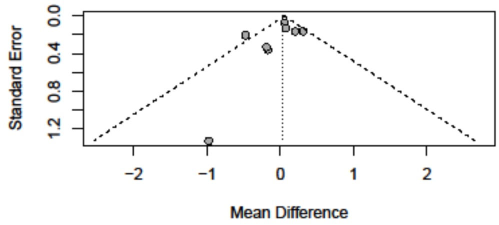

Fig. S1 (P) Funnel plot of BMI data in over 6 to 12 month old children (Egger's test for funnel plot asymmetry: p = 0.343)

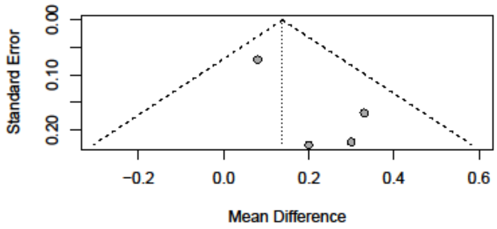

Fig. S1 (Q) Funnel plot of BMI data in over 12 month to under 3 year old children (Egger's test for funnel plot asymmetry: p = 0.130)

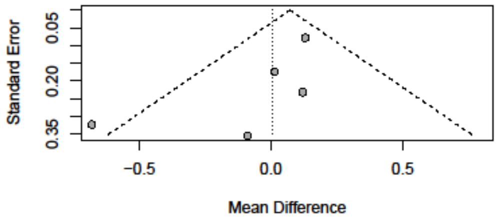

Fig. S1 (R) Funnel plot of BMI data in 3 year and older children (Egger's test for funnel plot asymmetry: p = 0.174)

Fig. S2 Forest plots illustrating the association of prenatal lifestyle interventions with (A) weight-for-age, (B) length-for-age, and (C) BMI z-scores in children

Study	Intervention Total	Intervention Mean	Intervention SD	Control Total	Control Mean	Control SD	MD	95%-CI
**Age = 1 to 2 months old**								
Delta Healthy Sprouts [2]	21	−1.07	1.46	29	−0.73	1.31	−0.34	[−1.13; 0.45]
GeliS [8]	861	0.89	1.35	849	0.92	1.28	−0.03	[−0.15; 0.09]
Kong et al. [13], 2014	18	−0.43	0.85	18	−0.13	0.72	−0.30	[−0.81; 0.21]
**Random effects model**	**900**			**896**			**−0.05**	**[−0.17; 0.07]**
Heterogeneity: I^2 = 0%								
**Age = 3 to 4 months old**								
Delta Healthy Sprouts [2]	20	−0.08	1.37	25	−0.42	1.31	0.34	[−0.45; 1.13]
GeliS [8]	865	0.90	1.27	845	0.93	1.19	−0.03	[−0.15; 0.09]
**Random effects model**	**885**			**870**			**−0.02**	**[−0.14; 0.09]**
Heterogeneity: I^2 = 0%								
**Age = 5 to 6 months old**								
Delta Healthy Sprouts [2]	18	0.37	1.43	26	0.41	1.27	−0.04	[−0.86; 0.78]
GeliS [2]	865	0.47	1.13	845	0.46	1.08	0.01	[−0.09; 0.11]
Kong et al. [13], 2014	15	−0.02	0.81	18	0.31	1.08	−0.33	[−0.98; 0.32]
LIMIT [14]	1071	0.30	1.13	1065	0.34	1.11	−0.04	[−0.13; 0.05]
ROLO [22]	138	0.93	1.59	142	0.65	1.00	0.28	[−0.03; 0.59]
UPBEAT [24]	321	0.20	1.08	322	0.29	1.12	−0.09	[−0.26; 0.08]
**Random effects model**	**2428**			**2418**			**−0.02**	**[−0.09; 0.05]**
Heterogeneity: I^2 = 10%								
**Age = Over 6 to 12 months old**								
Chiavaroli et al. [1], 2018	38	−0.15	0.98	23	−0.21	1.15	0.06	[−0.50; 0.62]
Delta Healthy Sprouts [2]	21	1.79	1.25	25	1.74	1.16	0.05	[−0.65; 0.75]
GeliS [8]	865	−0.05	1.03	848	−0.07	0.97	0.02	[−0.07; 0.11]
HAPPY [9]	38	0.33	0.98	40	0.53	0.92	−0.20	[−0.62; 0.22]
Healthy Moms [12]	51	0.37	0.74	52	0.51	0.94	−0.14	[−0.47; 0.19]
**Random effects model**	**1013**			**988**			**0.00**	**[−0.09; 0.09]**
Heterogeneity: I^2 = 0%								
**Age = 3 years and older**								
Chiavaroli et al. [1], 2018	33	0.34	0.86	24	0.43	0.58	−0.09	[−0.46; 0.28]
LIMIT [16]	1065	0.69	1.15	1056	0.64	1.15	0.05	[−0.05; 0.15]
**Random effects model**	**1098**			**1080**			**0.04**	**[−0.05; 0.14]**
Heterogeneity: I^2 = 0%								

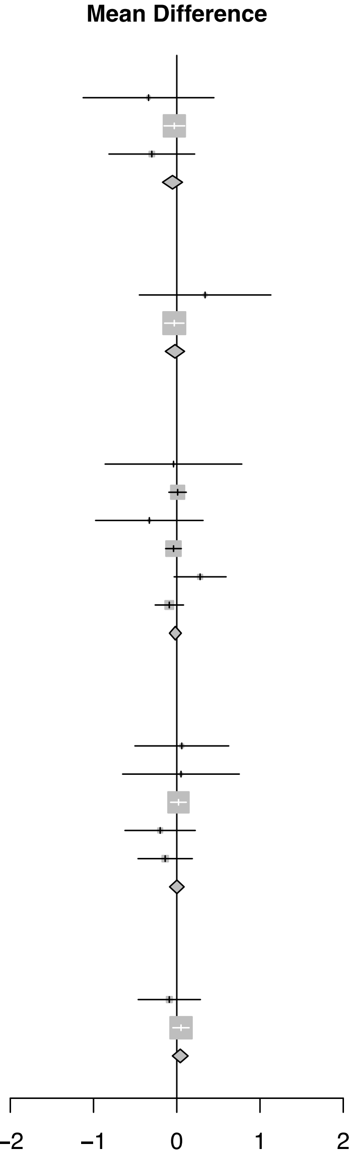

Fig. S2 (A) Forest plot illustrating the association of prenatal lifestyle interventions with weight-for-age z-score in children

Abbreviations: CI, confidence interval; MD, mean difference; SD, standard deviation

Study	Intervention Total	Intervention Mean	Intervention SD	Control Total	Control Mean	Control SD	MD	95%-CI
**Age = 1 to 2 months old**								
Delta Healthy Sprouts [2]	21	−1.46	1.61	28	−1.94	1.75	0.48	[−0.47; 1.43]
GeliS [8]	861	0.59	1.18	848	0.61	1.12	−0.02	[−0.13; 0.09]
Kong et al. [13], 2014	18	−0.65	1.16	18	−0.53	0.66	−0.12	[−0.74; 0.50]
**Random effects model**	**900**			**894**			**−0.02**	**[−0.12; 0.09]**
Heterogeneity: $I^2 = 0\%$								
**Age = 3 to 4 months old**								
Delta Healthy Sprouts [2]	20	−1.56	1.76	25	−2.39	2.35	0.83	[−0.37; 2.03]
GeliS [8]	865	0.96	1.17	844	1.03	1.14	−0.07	[−0.18; 0.04]
**Random effects model**	**885**			**869**			**0.17**	**[−0.61; 0.96]**
Heterogeneity: $I^2 = 53\%$								
**Age = 5 to 6 months old**								
Delta Healthy Sprouts [2]	18	−1.51	2.22	26	−1.98	2.47	0.47	[−0.93; 1.87]
GeliS [8]	864	0.58	1.08	845	0.60	1.09	−0.02	[−0.12; 0.08]
Kong et al. [13], 2014	15	0.05	0.96	18	0.12	1.12	−0.07	[−0.78; 0.64]
LIMIT [14]	1071	0.06	1.22	1065	0.11	1.28	−0.05	[−0.16; 0.06]
ROLO [22]	138	1.39	1.39	142	1.17	1.09	0.22	[−0.07; 0.51]
UPBEAT [24]	309	0.53	1.79	313	0.55	1.89	−0.02	[−0.31; 0.27]
**Random effects model**	**2415**			**2409**			**−0.02**	**[−0.09; 0.05]**
Heterogeneity: $I^2 = 0\%$								
**Age = Over 6 to 12 months old**								
Chiavaroli et al. [1], 2018	38	0.35	0.99	23	0.08	1.08	0.27	[−0.27; 0.81]
Delta Healthy Sprouts [2]	21	−0.41	2.98	25	−1.29	3.50	0.88	[−0.99; 2.75]
GeliS [8]	864	0.08	1.05	848	0.09	1.00	−0.01	[−0.11; 0.09]
Healthy Moms [12]	44	−0.15	0.97	41	0.20	1.31	−0.35	[−0.84; 0.14]
**Random effects model**	**967**			**937**			**−0.01**	**[−0.22; 0.19]**
Heterogeneity: $I^2 = 19\%$								
**Age = 3 years and older**								
Chiavaroli et al. [1], 2018	33	0.49	1.05	24	0.59	0.97	−0.10	[−0.63; 0.43]
LIMIT [16]	1065	0.11	1.10	1056	0.13	1.14	−0.02	[−0.12; 0.08]
**Random effects model**	**1098**			**1080**			**−0.02**	**[−0.12; 0.07]**
Heterogeneity: $I^2 = 0\%$								

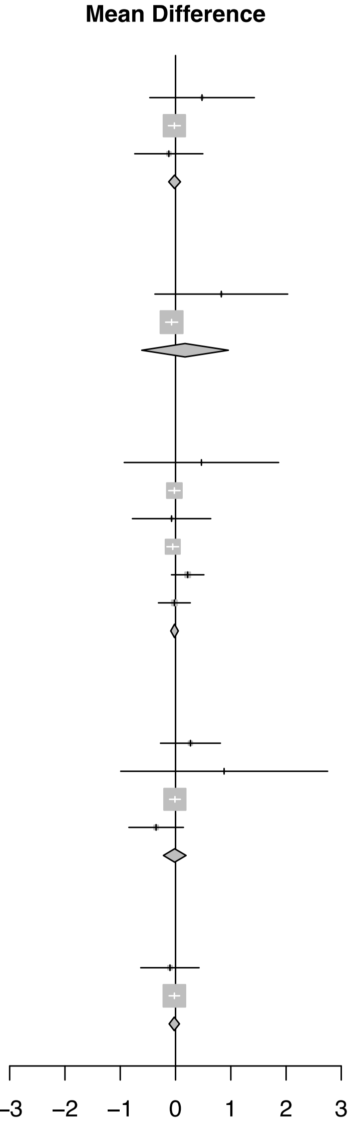

Fig. S2 (B) Forest plot illustrating the association of prenatal lifestyle interventions with length-for-age z-score in children

Abbreviations: CI, confidence interval; MD, mean difference; SD, standard deviation

Study	Intervention Total	Intervention Mean	Intervention SD	Control Total	Control Mean	Control SD	MD	95%-CI
**Age = 1 to 2 months old**								
GeliS [8]	861	0.48	1.24	848	0.49	1.23	−0.01	[−0.13; 0.11]
VIGA [25]	153	−0.15	0.89	136	−0.27	0.92	0.12	[−0.09; 0.33]
**Random effects model**	**1014**			**984**			**0.02**	**[−0.09; 0.14]**
Heterogeneity: $I^2 = 11\%$								
**Age = 3 to 4 months old**								
GeliS [8]	865	0.45	1.18	844	0.44	1.14	0.01	[−0.10; 0.12]
VIGA [25]	152	−0.09	0.94	136	−0.24	0.98	0.15	[−0.07; 0.37]
**Random effects model**	**1017**			**980**			**0.05**	**[−0.07; 0.16]**
Heterogeneity: $I^2 = 18\%$								
**Age = 5 to 6 months old**								
GeliS [8]	863	0.30	1.18	845	0.28	1.16	0.02	[−0.09; 0.13]
LIMIT [14]	1071	0.37	1.17	1065	0.38	1.16	−0.01	[−0.11; 0.09]
ROLO [22]	138	0.20	1.95	142	−0.01	1.05	0.21	[−0.16; 0.58]
UPBEAT [24]	317	−0.07	1.86	320	0.04	1.78	−0.11	[−0.39; 0.17]
VIGA [25]	153	0.28	0.89	137	0.14	0.93	0.14	[−0.07; 0.35]
**Random effects model**	**2542**			**2509**			**0.02**	**[−0.05; 0.08]**
Heterogeneity: $I^2 = 0\%$								
**Age = Over 6 to 12 months old**								
Chiavaroli et al. [1], 2018	38	−0.37	1.05	23	−0.26	1.16	−0.11	[−0.69; 0.47]
GeliS [8]	864	−0.03	1.18	848	−0.06	1.13	0.03	[−0.08; 0.14]
VIGA [25]	171	0.68	1.01	157	0.50	0.93	0.18	[−0.03; 0.39]
**Random effects model**	**1073**			**1028**			**0.06**	**[−0.04; 0.15]**
Heterogeneity: $I^2 = 0\%$								
**Age = Over 12 months to under 3 years old**								
LiPO [17]	82	0.06	1.06	75	−0.18	1.06	0.24	[−0.09; 0.57]
LIMIT [15]	1071	0.96	1.17	1065	0.92	1.06	0.04	[−0.05; 0.13]
VIGA [25]	152	0.89	0.92	136	0.64	1.05	0.25	[0.02; 0.48]
**Random effects model**	**1305**			**1276**			**0.13**	**[−0.02; 0.29]**
Heterogeneity: $I^2 = 46\%$								
**Age = 3 years and older**								
Chiavaroli et al. [1], 2018	33	0.09	0.80	24	0.12	0.67	−0.03	[−0.41; 0.35]
LIMIT [16]	1065	0.90	1.26	1056	0.79	1.17	0.11	[0.01; 0.21]
VIGA [25]	156	0.34	0.96	144	0.26	0.96	0.08	[−0.14; 0.30]
**Random effects model**	**1254**			**1224**			**0.10**	**[0.01; 0.19]**
Heterogeneity: $I^2 = 0\%$								

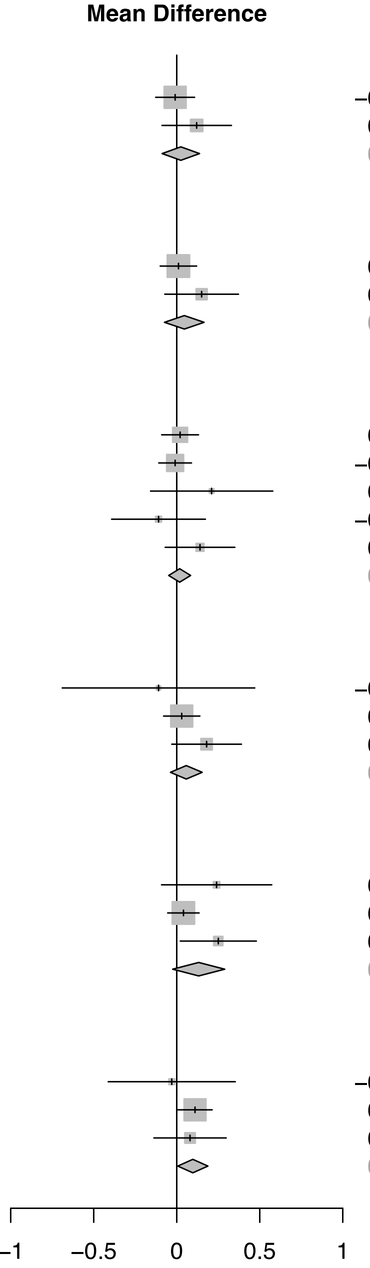

Fig. S2 (C) Forest plot illustrating the association of prenatal lifestyle interventions with BMI z-score in children

Abbreviations: CI, confidence interval; MD, mean difference; SD, standard deviation

References

1. Chiavaroli V, Hopkins S, Derraik J, Biggs J, Rodrigues RO, Brennan CH, Seneviratne SN, Higgins C, Baldi JC, McCowan LME *et al*: **Exercise in pregnancy: 1-year and 7-year follow-ups of mothers and offspring after a randomized controlled trial**. *Sci Rep* 2018, **8**(1):12915.
2. Thomson J, Goodman M, Tussing-Humphreys L, Landry A: **Infant growth outcomes from birth to 12 months of age: findings from the Delta Healthy Sprouts randomized comparative impact trial**. *Obesity Science and Practice* 2018, **4**(4):299-307.
3. Moholdt TT, Salvesen K, Ingul CB, Vik T, Oken E, Morkved S: **Exercise Training in Pregnancy for obese women (ETIP): study protocol for a randomised controlled trial**. *Trials* 2011, **12**:154.
4. Garnaes K, Morkved S, Salvesen K, Moholdt T: **Exercise training during pregnancy reduces circulating insulin levels in overweight/obese women postpartum: secondary analysis of a randomised controlled trial (the ETIP trial)**. *BMC Pregnancy Childbirth* 2018, **18**(1) (no pagination).
5. Parat S, Negre V, Baptiste A, Valensi P, Bertrand AM, Chollet C, Dabbas M, Altman JJ, Lapillonne A, Treluyer JM *et al*: **Prenatal education of overweight or obese pregnant women to prevent childhood overweight (the ETOIG study): an open-label, randomized controlled trial**. *Int J Obes (Lond)* 2019, **43**(2):362-373.
6. Rauh K, Gunther J, Kunath J, Stecher L, Hauner H: **Lifestyle intervention to prevent excessive maternal weight gain: mother and infant follow-up at 12 months postpartum**. *BMC Pregnancy Childbirth* 2015, **15**:265.
7. Phelan S, Hart C, Phipps M, Abrams B, Schaffner A, Adams A, Wing R: **Maternal behaviors during pregnancy impact offspring obesity risk**. *Exp Diabetes Res* 2011, **2011**:985139.
8. Hoffmann J, Günther J, Stecher L, Spies M, Geyer K, Raab R, Meyer D, Rauh K, Hauner H: **Infant growth during the first year of life following a pregnancy lifestyle intervention in routine care – findings from the cluster-randomised GeliS trial**. *Pediatric Obesity [accepted]* 2020.
9. McEachan R, Santorelli G, Bryant M, Sahota P, Farrar D, Small N, Akhtar S, Sargent J, Barber SE, Taylor N *et al*: **The HAPPY (Healthy and Active Parenting Programmme for early Years) feasibility randomised control trial: acceptability and feasibility of an intervention to reduce infant obesity**. *BMC Public Health* 2016, **16**:211.
10. Symons Downs D, Savage JS, Rivera DE, Smyth JM, Rolls BJ, Hohman EE, McNitt KM, Kunselman AR, Stetter C, Pauley AM *et al*: **Individually Tailored, Adaptive Intervention to Manage Gestational Weight Gain: Protocol for a Randomized Controlled Trial in Women With Overweight and Obesity**. *JMIR Res Protoc* 2018, **7**(6):e150.
11. Savage J, Hohman E, McNitt K, Pauley A, Leonard KS, Turner T, Pauli JM, Gernand AD, Rivera DE, Symons Downs D: **Uncontrolled Eating during Pregnancy Predicts Fetal Growth: The Healthy Mom Zone Trial**. *Nutrients* 2019, **11**(4).
12. Vesco K, Leo M, Karanja N, Gillman M, McEvoy C, King JC, Eckhardt CL, Smith KS, Perrin N, Stevens VJ: **One-year postpartum outcomes following a weight management intervention in pregnant women with obesity**. *Obesity (Silver Spring)* 2016, **24**(10):2042-2049.
13. Kong K, Campbell C, Wagner K, Peterson A, Lanningham-Foster L: **Impact of a walking intervention during pregnancy on post-partum weight retention and infant anthropometric outcomes**. *J Dev Orig Health Dis* 2014, **5**(3):259-267.
14. Dodd JM, McPhee AJ, Deussen AR, Louise J, Yelland LN, Owens JA, Robinson JS: **Effects of an antenatal dietary intervention in overweight and obese women on 6 month infant outcomes: follow-up from the LIMIT randomised trial**. *Int J Obes (Lond)* 2018, **42**(7):1326-1335.

15. Dodd JM, Louise J, Deussen AR, McPhee AJ, Owens JA, Robinson JS: **Prenatal Diet and Child Growth at 18 Months**. *Pediatrics* 2018, **142**(3).
16. Dodd J, Deussen A, Louise J: **Effects of an antenatal dietary intervention in women with obesity or overweight on child outcomes at 3-5 years of age: LIMIT randomised trial follow-up**. *Int J Obes (Lond)* 2020.
17. Tanvig M, Vinter C, Jorgensen J, Wehberg S, Ovesen P, Lamont RF, Beck-Nielsen H, Christesen HT, Jensen DM: **Anthropometrics and body composition by dual energy X-ray in children of obese women: a follow-up of a randomized controlled trial (the Lifestyle in Pregnancy and Offspring [LiPO] study)**. *PLoS One* 2014, **9**(2):e89590.
18. Aaltonen J, Ojala T, Laitinen K, Piirainen TJ, Poussa TA, Isolauri E: **Evidence of infant blood pressure programming by maternal nutrition during pregnancy: a prospective randomized controlled intervention study**. *J Pediatr* 2008, **152**(1):79-84, 84 e71-72.
19. Kolu P, Raitanen J, Puhkala J, Tuominen P, Husu P, Luoto R: **Effectiveness and Cost-Effectiveness of a Cluster-Randomized Prenatal Lifestyle Counseling Trial: A Seven-Year Follow-Up**. *PLoS One* 2016, **11**(12):e0167759.
20. Rono K, Stach-Lempinen B, Klemetti M, Kaaja R, Poyhonen-Alho M, Eriksson JG, Koivusalo SB, group R: **Prevention of gestational diabetes through lifestyle intervention: study design and methods of a Finnish randomized controlled multicenter trial (RADIEL)**. *BMC Pregnancy Childbirth* 2014, **14**:70.
21. Horan MK, McGowan CA, Gibney ER, Donnelly JM, McAuliffe FM: **Maternal diet and weight at 3 months postpartum following a pregnancy intervention with a low glycaemic index diet: results from the ROLO randomised control trial**. *Nutrients* 2014, **6**(7):2946-2955.
22. Horan M, McGowan C, Gibney E, Byrne J, Donnelly JM, McAuliffe FM: **Maternal Nutrition and Glycaemic Index during Pregnancy Impacts on Offspring Adiposity at 6 Months of Age--Analysis from the ROLO Randomised Controlled Trial**. *Nutrients* 2016, **8**(1).
23. Stafne S, Salvesen K, Romundstad P, Eggebo T, Carlsen SM, Morkved S: **Regular exercise during pregnancy to prevent gestational diabetes: a randomized controlled trial**. *Obstet Gynecol* 2012, **119**(1):29-36.
24. Patel N, Godfrey K, Pasupathy D, Levin J, Flynn AC, Hayes L, Briley AL, Bell R, Lawlor DA, Oteng-Ntim E *et al*: **Infant adiposity following a randomised controlled trial of a behavioural intervention in obese pregnancy**. *Int J Obes (Lond)* 2017, **41**(7):1018-1026.
25. Ronnberg A, Hanson U, Nilsson K: **Effects of an antenatal lifestyle intervention on offspring obesity - a 5-year follow-up of a randomized controlled trial.** *Acta Obstet Gynecol Scand* 2017, **96**(9):1093-1099.
26. Higgins J, Thomas J, Chandler J, Cumpston M: **Cochrane Handbook for Systematic Reviews of Interventions version 6.0 (updated July 2019)**. In.; 2019.
